# Therapeutic evaluation of [^212^Pb]Pb-AB001 and [^177^Lu]Lu-PSMA-617 in a mouse model of disseminated prostate cancer

**DOI:** 10.1007/s00259-025-07330-y

**Published:** 2025-05-21

**Authors:** Anna Julie Kjøl Høyvik, Monika Kvassheim, Li-Wei Ma, Elisabeth Wiig, Tiril Hillestad, Mona-Elisabeth Revheim, Rugile Liukaityte, Øyvind Bruland, Asta Juzeniene

**Affiliations:** 1 Department of Radiation Biology, Institute for Cancer Research, Oslo University Hospital, Oslo, 0379 Norway; 2 ARTBIO AS, Oslo, 0379 Norway; 3 Faculty of Medicine, Institute of Clinical Medicine, University of Oslo, Oslo, 0318 Norway; 4 Department of Physics and Computational Radiology, Division of Radiology and Nuclear Medicine, Oslo University Hospital, Oslo, 0379 Norway; 5 Department of Core Facilities, Institute for Cancer Research and Molecular Imaging, Oslo University Hospital, Oslo, 0379 Norway; 6 The Intervention Centre, Oslo University Hospital, Oslo, 0372 Norway; 7 Department of Oncology, Institute for Cancer Research, Oslo University Hospital, Oslo, 0379 Norway; 8 Faculty of Physics, University of Oslo, Oslo, 0318 Norway

**Keywords:** ^212^Pb, ^177^Lu, PSMA, Targeted alpha therapy, Disseminated prostate cancer model

## Abstract

**Background:**

Metastatic castration-resistant prostate cancer (mCRPC) frequently leads to bone and soft tissue metastases, leading to poor prognosis. The beta-emitting radioligand [^177^Lu]Lu-PSMA-617 targets the prostate-specific membrane antigen (PSMA) and may be less efficient against micrometastatic disease. The alpha-emitting radioligand [^212^Pb]Pb-AB001 could offer enhanced treatment by delivering high energy over a short range. This study compared the efficacy of [^212^Pb]Pb-AB001 and [^177^Lu]Lu-PSMA-617 in a mouse model of disseminated prostate cancer.

**Methods:**

Binding and internalisation of radioligands were evaluated in PC-3 PIP-luc cells. A mouse model was established by intracardiac injection of these cells. Treatments with 0.24‒1.0 MBq [^212^Pb]Pb-AB001 or 22‒66 MBq [^177^Lu]Lu-PSMA-617 were initiated 7 d post-cell inoculation. Metastatic burden was measured using bioluminescence imaging, and PSMA-targeted uptake was determined with [^18^F]F-PSMA-1007 µPET/µCT. Gamma-autoradiography evaluated [^212^Pb]Pb-AB001 distribution, and bone metastases were identified by radiography.

**Results:**

Both radioligands displayed comparable in vitro binding. In vivo studies revealed metastatic formation in clinically relevant organs. µPET/µCT demonstrated increased [^18^F]F-PSMA-1007 uptake in metastases, matching the bioluminescence imaging results. Focal [^212^Pb]Pb-AB001 distribution in the metastatic xenograft indicated heterogeneously distributed micrometastases in the organs. A median survival up to 47 d was achieved with [^212^Pb]Pb-AB001, compared to 25 d for controls and 27 d for [^177^Lu]Lu-PSMA-617. An activity-dependent reduction in bone metastases was observed for [^177^Lu]Lu-PSMA-617, while no bone lesions were detected in [^212^Pb]Pb-AB001-treated mice.

**Conclusion:**

[^212^Pb]Pb-AB001 showed significant efficacy against micrometastases and advantages over [^177^Lu]Lu-PSMA-617 in preventing or treating early bone metastases for the investigated injected activities. This implies clinical potential for treating mCRPC, including patients at risk of early metastatic disease, but further studies including dosimetry and toxicity analyses are required with regards to activity levels.

**Supplementary Information:**

The online version contains supplementary material available at 10.1007/s00259-025-07330-y.

## Introduction

Castration-resistant prostate cancer (CRPC) develops in 10‒20% of prostate cancer patients, with over 84% presenting with metastases at diagnosis [1, 2]. Among nonmetastatic CRPC patients, 56% develop metastases within 3 years [3]. Metastatic CRPC (mCRPC) has a high prevalence of bone metastases, affecting 80‒90% of patients, and frequent metastases in lymph nodes (20‒65%), liver (10‒40%) and lungs (10‒20%) [3, 4]. Over 90% of mCRPC patients exhibit detectable levels of circulating tumour cells (CTCs) in the blood, and elevated CTC levels are associated with poor prognosis and increased prevalence of bone metastases [5–7]. Despite advances in therapy, mCRPC remains incurable and patients experience inevitable relapses.

**Fig. 1:**
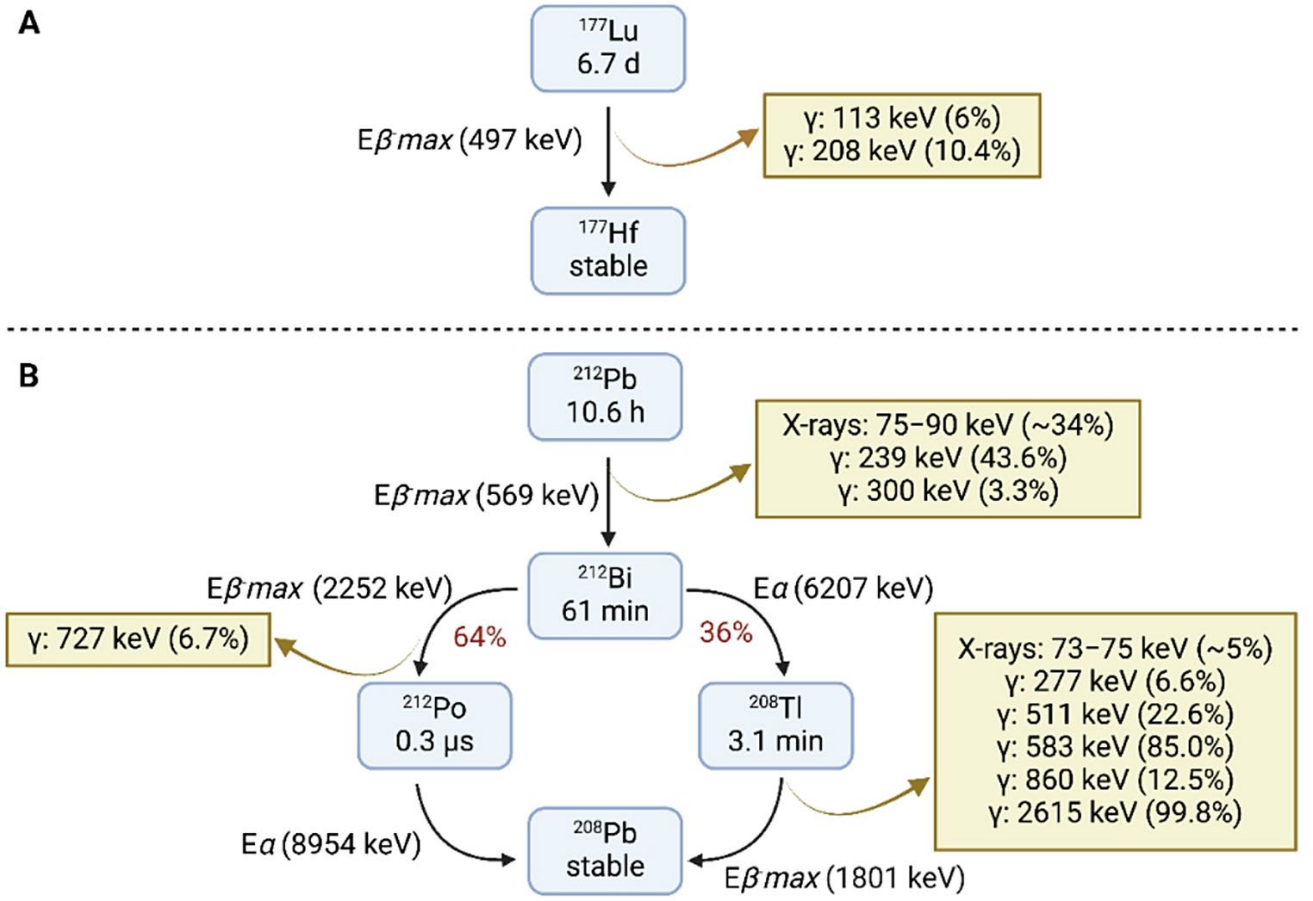
Simplified decay schemes of (**A**) ^177^Lu and (**B**) ^212^Pb. E*β*^*−*^*max* and E*α* represent the highest energy available for beta and alpha emissions, respectively. Emissions with energy > 70 keV and intensities > 5% are included. Energies are not corrected for branching. Data was extracted from the National Nuclear Data Center, and the figure was created in BioRender

In 2022, the prostate-specific membrane antigen (PSMA)-targeting radioligand [^177^Lu]Lu-PSMA-617 (Pluvicto) received approval for the treatment of advanced mCRPC after demonstrating improved overall survival and radiographic progression-free survival [8]. Lutetium-177 (t_1/2_ = 6.7 d) emits beta particles (E_*βmax*_ = 0.497 MeV) with a tissue penetration range of 0.2–1.7 mm, leading to a low linear energy transfer (LET) of ~ 0.2‒2 keV/µm (Fig. 1A) [9]. Moreover, the 31 h effective half-life of [^177^Lu]Lu-PSMA-617 for whole-body clearance is significantly shorter than the physical half-life of ^177^Lu [10], resulting in substantial activity excretion through biological clearance. A recent study reported poorer treatment responses to [^177^Lu]Lu-PSMA-617 in patients with elevated CTC levels [11]. This may be attributed to insufficient energy delivery at the cellular level, as beta particles disperse their energy over a longer range compared to alpha particles, leading to a lower probability of causing clustered DNA damage within a single cell.

Alpha particles have a high LET (50‒230 keV/µm) and a short range in tissue (< 100 μm), increasing the probability of cytotoxic effects to be confined to targeted cells. Targeted alpha therapy with actinium-225 (t_1/2_ = 9.9 d) has shown promise for mCRPC preclinically and clinically, but it remains limited by concerns over toxicity and production capacity [12–15]. Thus, a ^212^Pb-based approach has become a feasible alternative in this therapeutic space. Lead-212 (t_1/2_ = 10.6 h) is being investigated for the treatment of metastatic cancers because of its appropriate physical half-life and emission of alpha particles via its daughter radionuclides (E_*α*_ = 6.2‒9.0 MeV, Fig. 1B) [16, 17]. The physical half-life aligns well with the biological half-life of small molecule ligands, offering rapid uptake and energy deposition to the tumour within a few hours [18, 19]. A recent Phase 0 study reported an 8 h effective whole-body half-life for the PSMA-targeting radioligand [^212^Pb]Pb-AB001 (formerly [^212^Pb]Pb-NG001) [20], allowing a substantial fraction of the total decay to occur before biological clearance. Additionally, ^212^Pb can be imaged via single photon emission computed tomography (SPECT) due to the emission of X-rays and gamma rays during the decay to ^212^Bi, allowing for the quantitative assessment of nuclide uptake in tumours (Fig. 1B) [18, 20–22]. Preclinical and early clinical studies support the potential applicability and safety of targeted alpha therapy with ^212^Pb (NCT05153772, NCT05725070) [19, 23–29].

In previous preclinical studies, [^212^Pb]Pb-AB001 has demonstrated therapeutic efficacy in subcutaneous C4-2 and PC-3 PIP xenografts [26, 27]. While this model enables uptake and tumour monitoring, it does not recapitulate metastasis in mCRPC. Therefore, in this study, we evaluated the therapeutic efficacy of [^212^Pb]Pb-AB001 and [^177^Lu]Lu-PSMA-617 in a metastatic prostate cancer model. The PSMA-targeting ligand AB001 (previously NG001) is structurally similar to PSMA-617, sharing the same glutamate-urea-based PSMA-binding motif but differing in its chelator and linker design [30].

## Materials and methods

### Preparation of [^212^Pb]Pb-AB001

High-purity ^212^Pb (> 99.99%) was produced using a single-chamber diffusion generator with a ^228^Th source (Oak Ridge National Laboratory) [31, 32]. The eluted ^212^PbCl_2_ was pH-adjusted to 5–6 with 5 M NaOAc, and the AB001 ligand (MedKoo Biosciences) was added to a specific activity of 2 MBq/µg (3.3 MBq/nmol). Radiolabelling, quality control and radioactive measurements followed established protocols [27]. For in vivo use, bovine serum albumin (Merck Norway AS, Oslo, Norway) was added to a final concentration of 0.16 µg/µL, followed by dilution in 0.9% NaCl and sterile filtration.

### Preparation of [^177^Lu]Lu-PSMA-617

The PSMA-617 ligand (MedKoo Biosciences) was prepared as described by Stenberg et al. [33]. Briefly, 10 µl of 5 M NaOAc was preheated with the PSMA-617 ligand for 5 min at 90 °C and 650 rpm before adding no-carrier-added ^177^LuCl_3_ in 0.04 M HCl (EndolucinBeta, ITM) to a specific activity of 89 MBq/µg (133 MBq/nmol). The pH was adjusted to 5‒6 with 5 M NaOAc or 0.1 M HCl. Radiochemical purities > 95% were confirmed, and injection solutions were prepared as described above. ^177^Lu-activity was measured using a CRC-25R or CRC-55tR radioisotope dose calibrator (Capintec Inc.) with a calibration factor of 450 × 10.

### Cell line

PC-3 PIP cells, kindly provided by Dr. Martin Pomper (John Hopkins University School of Medicine, Baltimore, MD, USA), were maintained as previously described [27]. Cells were transduced with RediFect Red-FLuc-GFP Lentiviral Particles (Perkin Elmer, Waltham, MA, USA) according to the manufacturer’s protocol and sorted via fluorescence-activated cell sorting to generate the PC-3 PIP-luc cell batch.

### Ligand reactive fraction

The ligand reactive fraction (LRF) was assessed by incubating 9‒12 × 10^6^ PC-3 PIP-luc cells in 200 µl 0.5% w/v BSA in PBS with 1 ng of the PSMA-targeted radioligand for 1 h. Non-specific binding was determined by pre-incubating the cells with 10 µg of unlabelled cold ligand for 15 min. Total binding was measured using a gamma counter before and after washing the cells three times with 0.5% BSA in PBS. Internalisation was assessed by incubating the samples for 10 min in 50 mM glycine stripping buffer, followed by three washes in 0.5% w/v BSA in PBS to remove surface-bound radioligands. Specific binding was calculated by subtracting non-specific from total binding, while internalised activity was expressed as a percentage of added or specific cell-bound activity.

### Research animals

Experiments were conducted with 131 male Hsd: Athymic Nude-Foxn1^nu^ mice (6‒8 w old, 25–35 g) bred at the Department of Comparative Medicine at the Norwegian Radium Hospital (Oslo University Hospital, Oslo, Norway) or purchased from Inotiv (Horst, Netherlands). Mice were housed under pathogen-free conditions with food and water ad libitum and a 12 h–12 h light-dark cycle. The studies were approved by the Institutional Committee on Research Animal Care and the Norwegian Food Safety Authority (FOTS-ID 30148 and 30625; Brumunddal, Norway).

### Disseminated xenografts

For in vivo procedures, anaesthesia using 3% sevoflurane and oxygen at 1.0 L/min or 2% isoflurane and oxygen at 350 ml/min was used, and eye ointment was added to prevent corneal drying. Dissemination was initiated by injecting 2.5 × 10^6^ PC-3 PIP-luc cells into the left cardiac ventricle. Successful intracardiac (IC) injection was assessed by the pulsating bright red blood in the syringe following whole-body cell distribution confirmed by 2D bioluminescence imaging (BLI) using the In Vivo Imaging System (IVIS) Spectrum Xenogen (Perkin Elmer) 10–15 min post intraperitoneal (IP) injection of 200 µl of IVISBrite D-luciferin (15 µg/µl, Perkin Elmer).

### Bioluminescent imaging

The in vitro linearity between cell number and bioluminescence was evaluated in a 96-well plate (0–10⁷ cells/well) 10–15 min after adding IVISBrite D-luciferin (150 µg/µL). For ex vivo studies, mice were injected IP with 200 µl IVISBrite D-luciferin (15 µg/µl) following sacrifice after 5–10 min. Organs were transferred to a petri dish following BLI of the organs. Metastatic disease burden was monitored in vivo using 2D or 3D BLI 10–15 min post IP injection with 200 µl of IVISBrite D-luciferin (15 µg/µl). All imaging procedures were performed using the IVIS Spectrum Xenogen (Perkin Elmer), IVIS Spectrum CT (Perkin Elmer) or an ultra-high-resolution VECTor/OI-CT Imaging system (MILabs, Houten, Netherlands). Images were analysed using the Living Image software (Perkin Elmer) or the Imalytics 3.1 software (iThera Medical, Munich, Germany).

### Preclinical μPET/µCT

Static micro positron emission tomography (µPET)/micro computed tomography (µCT) scans (30 min) were acquired on anesthetized mice on days 0, 9 and 21, using a small animal PET-scanner (MR solutions PET/CT benchtop, Guildford, UK) 2 h post intravenous (IV) injection of 4‒6 MBq ^18^F-PSMA-1007 (Norwegian Medical Cyclotron Center, Oslo Norway). The spatial resolution with 3D OSEM µPET/µCT was 0.7 mm, with a sensitivity of 7.5%. Computed tomograpy was acquired at 40 kVP. The images were reconstructed with a 3D OSEM algorithm (MR solutions) on 0.84 × 0.84 × 0.84 mm^3^ voxels and prepared for publication using Slicer version 5.6.0.

### Preclinical SPECT/µCT

Single-photon emission computed tomography/µCT imaging (15 min) was acquired on anesthetised animals 1 h after IV injection of 1 MBq [^212^Pb]Pb-AB001, using a high-energy ultra high sensitivity mouse 3.80 mm collimator and a VECTor/µCT 6 (MILabs), with a spatial resolution of 0.9 mm and sensitivity of 7.2%. Computed tomography was acquired at 50 kVP with 49 CTDIvol. Images were reconstructed from energy windows of 40% at 79 keV and 20% at 239 keV, with adjacent dual scatter windows of 10% and 5%, respectively. Reconstructions were performed with the MILabs reconstruction algorithm SROSEM, with 4 iterations and 16 subsets on 0.8 mm^3^ voxels and a Gaussian Blurring post filter of 1.2 mm. Triple energy window scatter correction with a background weight of 2 and attenuation correction by CT were applied. Images were prepared for publication using Slicer version 5.6.0.

### Gamma-autoradiography

Gamma autoradiography (Fujifilm BAS-5000, Tokyo, Japan) was used to visualise [^212^Pb]Pb-AB001 distribution at sub-tissue levels. One metastasis-bearing mouse (IC inoculation 7 d prior) and two negative controls (no cancer cells injected) were sacrificed 2 h post IV administration of 1.01 ± 0.06 MBq [^212^Pb]Pb-AB001. Organs were harvested, embedded in 99% isopentane on dry ice, and cryosectioned (CryoStar NX70, Thermo Fisher Scientific). Lungs and liver were sectioned in 10 μm and femurs in 20 μm. Three slices per organ were placed on a Fuji imaging plate (25 μm resolution) and imaged for 12 h. Image analysis (Multi Gauge 3.2) included decay correction of photostimulated luminescence from scan start to euthanasia for direct comparison between mice.

### Therapeutic efficacy

Mice were assigned to treatment groups 6 d post IC inoculation based on BLI-confirmed metastases in comparable organs and similar levels of metastatic burden (p/s/cm^2^/sr, Fig. S4). The efficacy of [^212^Pb]Pb-AB001 and [^177^Lu]Lu-PSMA-617 was assessed in four and two independent studies, respectively. Treatments were administered IV with 0.25 ± 0.02, 0.49 ± 0.03 or 1.04 ± 0.003 MBq [^212^Pb]Pb-AB001 (0.07‒0.31 nmol), or 22.0 ± 0.01, 42.9 ± 1.39 or 66.0 ± 0.03 MBq [^177^Lu]Lu-PSMA-617 (0.17‒0.50 nmol). The control mice received 100 µl 0.9% NaCl.

### Radiography

At the humane or experimental endpoint, X-ray imaging was performed on mice treated with 0.5 or 1.0 MBq [^212^Pb]Pb-AB001 or 22, 43 or 66 MBq [^177^Lu]Lu-PSMA-617 using a MultiRad 225 irradiation system with a 10 cm × 15 cm CMOS detector (Precision X-Ray, Madison, WI, USA). Images (2 s exposure, 32 kV, 1 mA) were acquired in anterior and posterior positions, and lesions were identified using the medical imaging viewer Horos (Horos Project v3.3.6).

### Statistical analysis

SigmaPlot 15.0 was used for the statistical analyses (Systat Software, Inc., San Jose, CA, USA). Group comparisons were performed by one-way ANOVA followed by multiple comparisons or t-tests, and survival was analysed via Kaplan-Meier curves and log-rank tests. A *p*-value < 0.05 was considered significant.

## Results

### Establishment and characterisation of the disseminated model

PC-3 PIP cells were transduced with a GFP-luciferase-expressing lentiviral vector and sorted using flow cytometry (Fig. S1). The transduction was stable throughout the study period (Fig. S2), and a linear relationship was observed between cell number and BLI (Fig. S3). After IC inoculation of 2.5 × 10^6^ PC-3 PIP-luc cells, 2D BLI of mice detected CTC in the cardiovascular system, with increased signals observed in the kidneys, liver, testes and brain (Fig. S4).

**Fig. 2:**
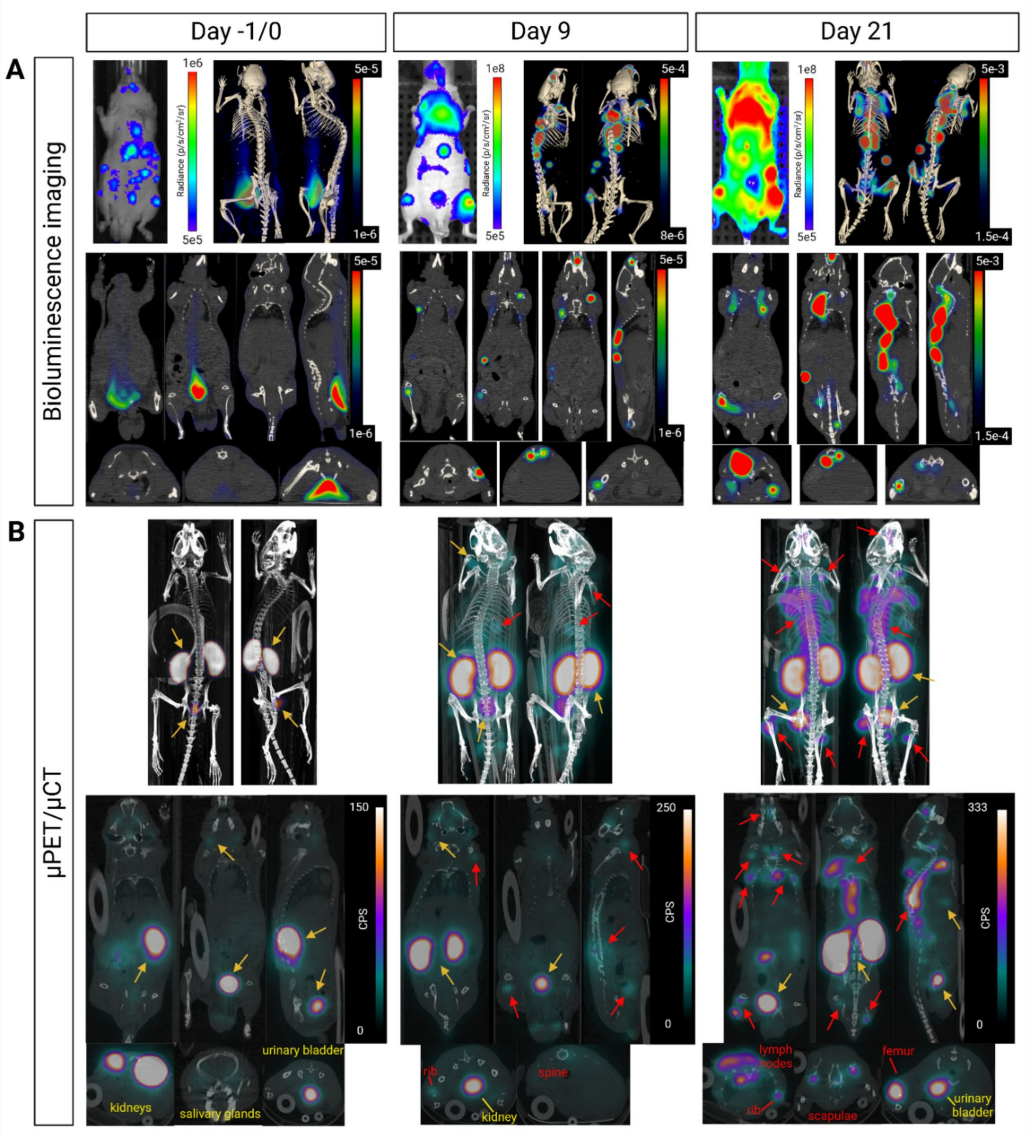
Representative bioluminescence and µPET/µCT images of a control xenograft. The mouse received 2.5 × 10^6^ PC-3 PIP-luc cells intracardially on day - 7 and 0.9% NaCl on day 0. (**A**) 2D and 3D bioluminescence imaging was performed on day - 1, illustrating localisation of PC-3 PIP-luc cells and metastatic burden over time. (**B**) 30 min µPET/µCT acquisition was performed on days 0, 9 and 21, 2 h after intravenous administration of 4‒6 MBq [^18^F]F-PSMA-1007. Red arrows denote uptake of [^18^F]F-PSMA-1007 in metastatic sites, while yellow arrows indicate uptake in nonmetastatic tissues. The images include a colour-mapped scale demonstrating increased metastatic burden and uptake of [^18^F]F-PSMA-1007 up to day 21

One day before radioligand administration (day - 1), metastatic foci and CTC were detected by 2D and 3D BLI (Fig. 2A and S4). The cell signal intensity decreased by 90% from day - 7 (cell inoculation day) to day - 1 (*p* < 0.001, Fig. S4B), resulting in a remaining cell population of approximately 0.25 × 10^6^ cells. On day 0, µPET/µCT and SPECT/µCT did not reveal [^18^F]F-PSMA-1007 or [^212^Pb]Pb-AB001 uptake, as the lesions were too small. Moreover, the SPECT/µCT images were limited by noise, indicating a need for model optimisation of preclinical ^212^Pb SPECT/µCT in this model (Fig. S5C). By days 9 and 21, [^18^F]F-PSMA-1007 uptake was evident in clinically relevant sites, including the skeleton (spine, femurs, tibia, scapula and ribs) and soft tissues (liver, lungs and lymph nodes) of the control mice (Fig. 2B). The µPET/µCT scan correlated with 2D and 3D BLI (Fig. 2 [red arrows]) and was consistent with the organs harbouring metastases at the humane endpoint, which revealed metastatic take at 100% in the femurs, 57% in the skull, 71% in the lungs, and 57% in the liver (Table S1). Nonmetastatic uptake of [^18^F]F-PSMA-1007 was identified in the kidneys, gallbladder, urinary bladder and salivary glands (Fig. 2B [yellow arrows]).

### Binding and internalisation

**Fig. 3:**
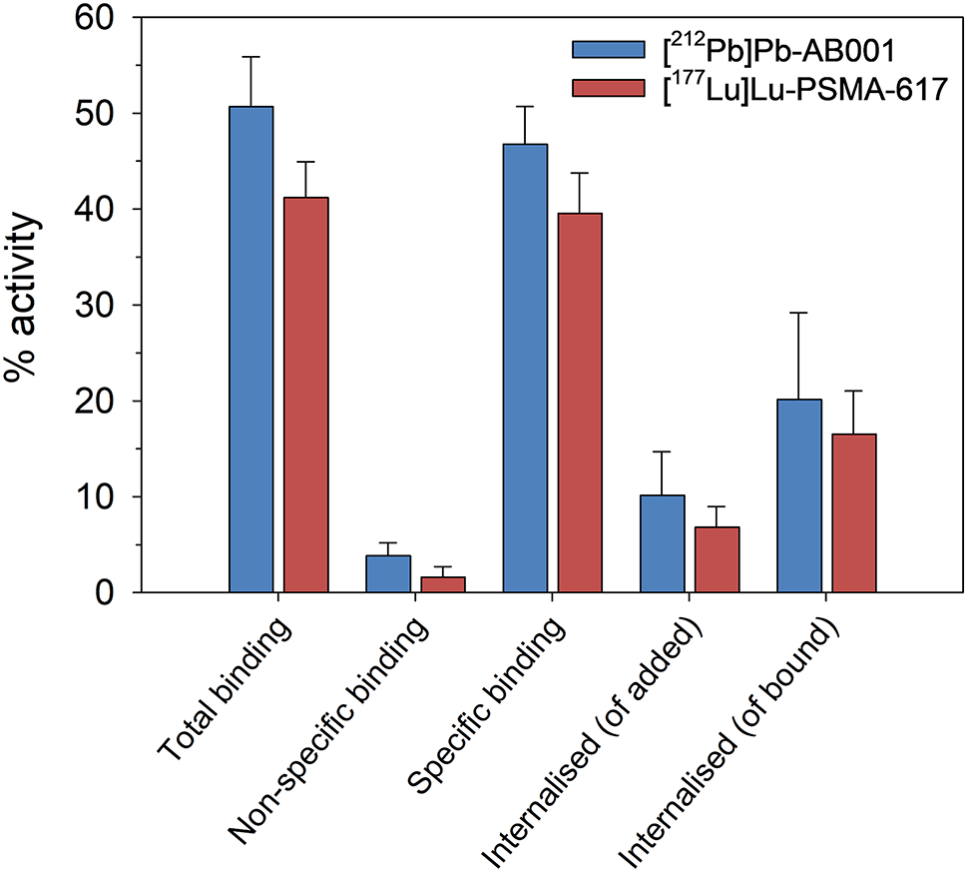
Binding and internalisation of [^212^Pb]Pb-AB001 and [^177^Lu]Lu-PSMA-617 in PC-3 PIP-luc cells. Percentage total binding (of added activity), non-specific binding (blocked), specific binding, internalised (of added activity) and internalised (of specifically bound activity) [^212^Pb]Pb-AB001 or [^177^Lu]Lu-PSMA-617 in PC-3 PIP-luc cells. The data are presented as the average ± standard deviation of independent experiments (*n* = 4 for [^212^Pb]Pb-AB001, *n* = 3 for [^177^Lu]Lu-PSMA-617). Statistical differences between groups were analysed using one-way ANOVA followed by multiple comparisons

The radioligands exhibited comparable LRF levels (*p* > 0.05, Fig. 3). The percentage of specifically bound radioligand was 46.8 ± 3.9% for [^212^Pb]Pb-AB001 and 39.6 ± 4.2% for [^177^Lu]Lu-PSMA-617, of which the internalised proportion was 20.2 ± 9.0% and 16.4 ± 4.5%, respectively.

### Autoradiography

**Fig. 4:**
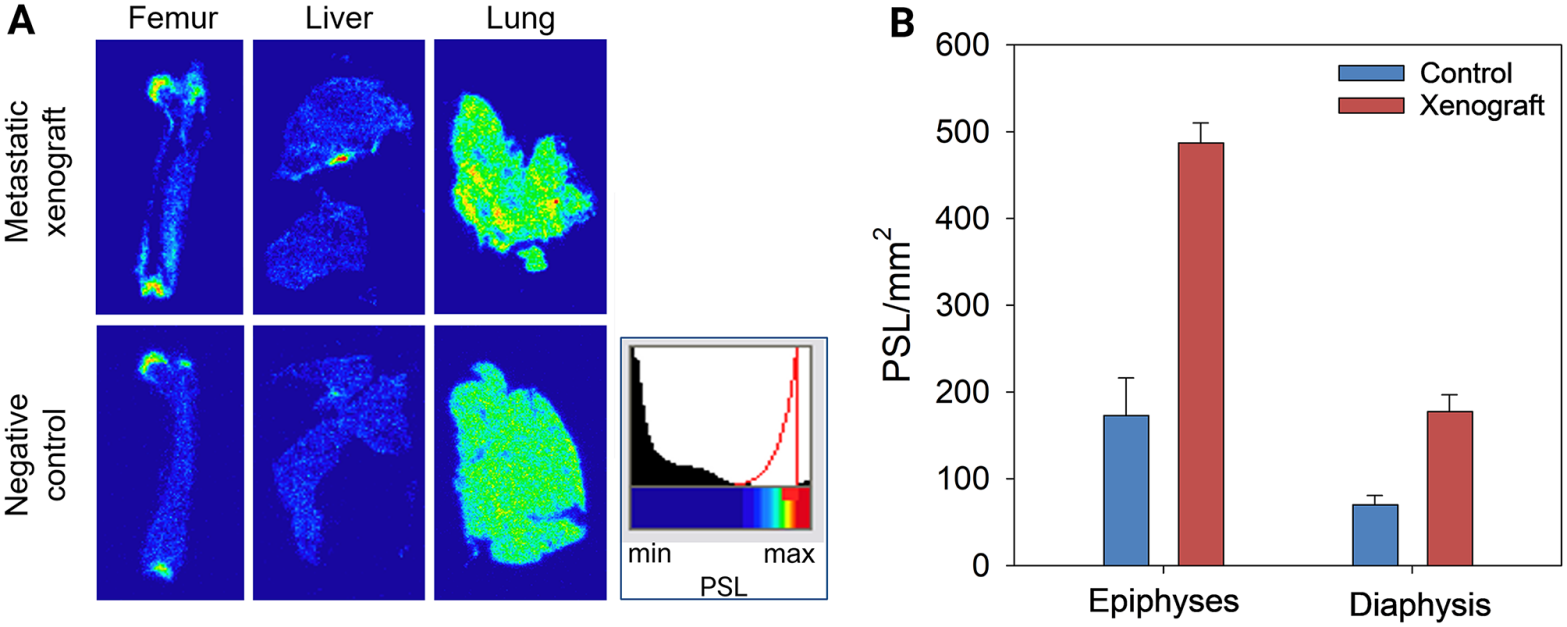
Representative gamma-autoradiography analysis. (**A**) Overnight gamma-autoradiography (12 h exposure) of the femurs, liver and lungs from a metastatic xenograft and negative controls euthanised 2 h post administration of 1 MBq [^212^Pb]Pb-AB001. (**B**) Uptake of [^212^Pb]Pb-AB001, measured in photostimulated luminescence (PSL) per mm^2^, in the epiphyses and diaphysis of the femurs from negative controls (*n* = 2) and a metastatic xenograft (*n* = 1). The statistical difference in PSL/mm^2^ values between the controls and the xenograft was assessed using a t-test

Ex vivo gamma-autoradiography was performed on organs from mice sacrificed 2 h after injection of 1.0 MBq [^212^Pb]Pb-AB001. In the controls, gamma-autoradiography indicated a homogeneous distribution of [^212^Pb]Pb-AB001 in the liver and lungs (Fig. 4A). By contrast, the metastatic xenograft displayed focal regions with higher intensity (red hotspots) in the femoral epiphyses, lungs and liver, indicating localised [^212^Pb]Pb-AB001 accumulation in micrometastases heterogeneously distributed in the organs (Fig. 4A). In the femurs, the metastatic xenograft exhibited 2.8- and 2.5-fold higher distribution of [^212^Pb]Pb-AB001 in the epiphyses and diaphysis, respectively, compared to the control (*p* < 0.01, Fig. 4B).

### Therapeutic efficacy of [^212^Pb]Pb-AB001 and [^177^Lu]Lu-PSMA-617

**Fig. 5:**
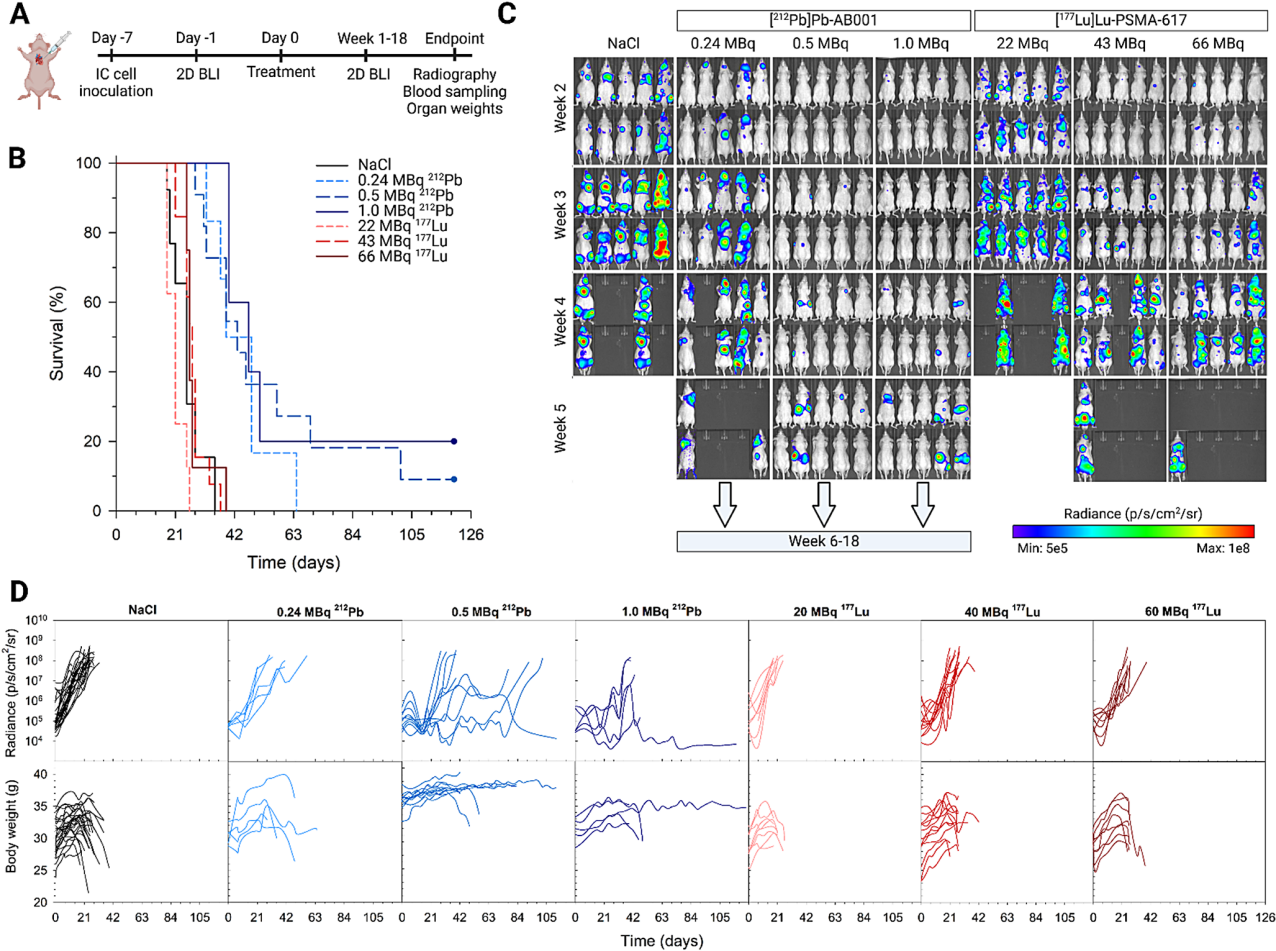
Experimental design and therapeutic efficacy of [^212^Pb]Pb-AB001 and [^177^Lu]Lu-PSMA-617. (**A**) Experimental design of therapy studies. (**B**) Kaplan-Meier survival curves for the control (NaCl) mice and mice treated with [^212^Pb]Pb-AB001 (0.24, 0.5 and 1.0 MBq) or [^177^Lu]Lu-PSMA-617 (22, 43 and 66 MBq). Survival analysis was followed by pairwise comparisons using a log-rang test. (**C**) Representative 2D bioluminescence images of disseminated xenografts demonstrating metastatic burden over time. Arrows indicate survival and further ongoing study for these groups (complete data shown in Fig. S6). (**D**) Longitudinal plots demonstrating whole-body metastatic progression (upper) and body weight changes (lower) of individual mice

Across four independent experiments, the control mice exhibited rapid disease progression, with a median survival of 25 d (Fig. 5C‒D; Table 1). Tumour growth delay was observed post treatment with 43‒66 MBq [^177^Lu]Lu-PSMA-617 (Fig. 5C and Table S3), but no survival improvement over the controls at any studied activity (*p* > 0.05, Fig. 5B; Table 1). The median survival was 21, 27 and 26 d for mice treated with 22, 43 and 66 MBq of [^177^Lu]Lu-PSMA-617, respectively, resulting in a maximum therapeutic index (TI) of 1.1 (Table 1). By contrast, all studied activities of [^212^Pb]Pb-AB001 delayed overall metastatic growth (Fig. 5B and Table S3), and prolonged survival significantly compared to the controls (*p* ≤ 0.002) and any of the [^177^Lu]Lu-PSMA-617 treated groups (*p* < 0.04, Table S2). At 0.24, 0.5 and 1 MBq [^212^Pb]Pb-AB001, the resulting median survivals were 43.5, 43 and 47 d, corresponding to TIs of 1.7, 1.7 and 1.9, respectively (Table 1).

**Table 1:** Summary of injected activities, median survivals and therapeutic indexes for athymic nude mice bearing PC-3 PIP-luc metastases treated with [^212^Pb]Pb-AB001 or [^177^Lu]Lu-PSMA-617. The therapeutic index (TI) was calculated as the ratio of the median survival of each treatment group to that of the control group. Statistical significance was determined using a log-rank test with multiple pairwise comparisons (Holm-Sidak method)

Treatment group	*N* mice (*N* studies)	Injected activity	Injected ligand (nmol/mouse)	Median survival	TI	*p*-value relative to the control
Control	26 (4)	**-**		25	1	**-**
0.25 MBq [^212^Pb]Pb-AB001	6 (1)	0.24 ± 0.02 MBq	0.07	43.5	1.7	0.002
0.5 MBq [^212^Pb]Pb-AB001	11 (2)	0.49 ± 0.03 MBq	0.15	43	1.7	< 0.001
1.0 MBq [^212^Pb]Pb-AB001	5 (1)	1.04 ± 0.003 MBq	0.30	47	1.9	0.001
20 MBq [^177^Lu]Lu-PSMA-617	8 (1)	22.0 ± 0.01 MBq	0.17	21	0.8	0.132
40 MBq [^177^Lu]Lu-PSMA-617	13 (2)	42.9 ± 1.39 MBq	0.32	27	1.1	0.891
60 MBq [^177^Lu]Lu-PSMA-617	8 (1)	66.0 ± 0.03 MBq	0.50	26	1	0.891

**Fig. 6:**
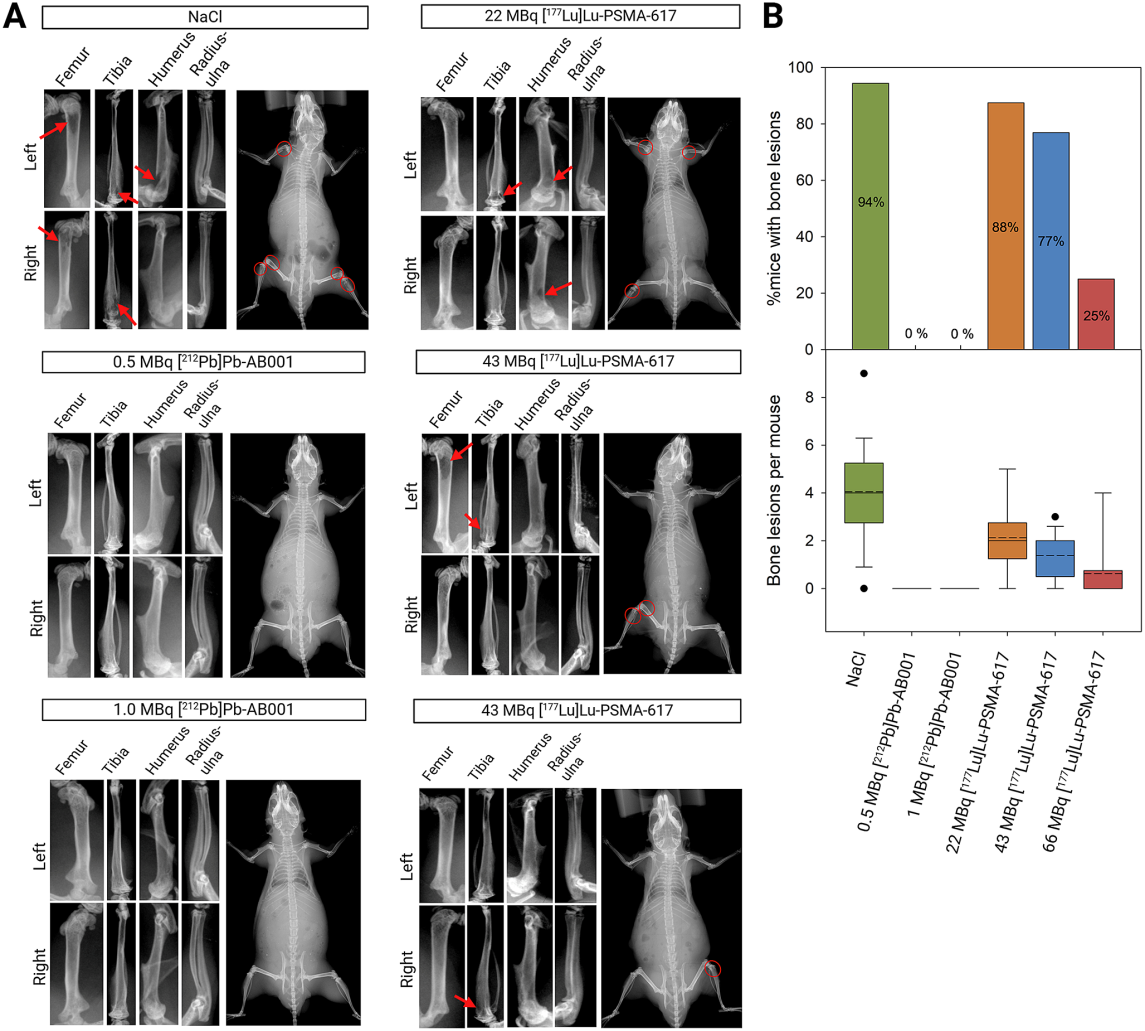
Comparative efficacy of [^212^Pb]Pb-AB001 and [^177^Lu]Lu-PSMA-617 in treating bone metastases. (**A**) Representative X-ray images showing bone lesions in the femur, tibia, humerus, and radius-ulna of mice receiving NaCl, 0.5 or 1.0 MBq [^212^Pb]Pb-AB001, or 22, 43 or 66 MBq [^177^Lu]Lu-PSMA-617. Red arrows and circles indicate osteolytic bone lesions. (**B**) Percentage of mice with bone lesions at the humane endpoint (upper panel), and the average number of lesions per mouse (lower panel). The bottom of the boxes represents the 25th percentiles, solid lines represent the medians, dashed lines represent the mean, top of the boxes represent the 75th percentiles and whiskers represent the 5th and 95th percentiles. Statistical differences between groups were analysed using one-way ANOVA followed by multiple comparisons

At the humane or experimentally defined endpoint, radiography of the control and treated mice (0.5 and 1 MBq [^212^Pb]Pb-AB001 or 22, 43 and 66 MBq [^177^Lu]Lu-PSMA-617) revealed osteolytic bone lesions (Fig. 6A). In the control group, 94% of the mice had bone metastases in the femurs, tibia, humerus and radius-ulna, averaging 4.1 lesions per mouse (Fig. 6). Mice treated with [^177^Lu]Lu-PSMA-617 displayed an activity-dependent reduction in bone metastases, with 88%, 77%, and 25% of mice exhibiting bone metastases with the average number of lesions per mouse decreasing from 2.1 to 1.5 and 0.7 post treatment with 22, 43, and 66 MBq, respectively (Fig. 6B). By contrast, [^212^Pb]Pb-AB001 treated mice had no detectable bone metastases (Fig. 6), and were euthanized due to visceral metastases, precluding longer observation for bone metastases.

Body or organ weights (lungs, salivary glands, femurs, liver, spleen and kidneys) showed no significant difference between the control and treatment groups (*p* > 0.05, Fig. S7A). Serum parameters, including glutamic pyruvic transaminase, glutamic oxaloacetic transaminase and alkaline phosphatase, showed no signs of liver toxicity (*p* > 0.05, Fig. S7B). Amylase or urea values showed no elevated levels compared to the control (*p* > 0.05), indicating no pancreatic or kidney disease, respectively. Haematological analysis revealed no abnormalities in white or red blood cell count, haemoglobin or platelet values across treatment groups compared with the control (*p* > 0.05, Fig. S7C).

## Discussion

This preclinical study is the first to report on bone lesion status following PSMA-targeted radioligand therapy. Our findings demonstrate the effective targeting of [^212^Pb]Pb-AB001 to metastatic foci (Fig. 4), significant survival benefits, metastatic growth delay and absence of bone metastases after [^212^Pb]Pb-AB001 treatment (Figs. 5 and 6). By contrast, [^177^Lu]Lu-PSMA-617 did not significantly prolong survival at any activity level tested compared to the control (*p* < 0.05, Fig. 5), consistent with previous studies documenting its limited efficacy in micrometastatic models (Table S4) [11, 14, 25, 34]. Nonetheless, [^177^Lu]Lu-PSMA-617 demonstrated a slight delay in metastatic progression and an activity-dependent reduction in bone lesions, though less effectively than [^212^Pb]Pb-AB001 for the administered activities used (Fig. 6 and Table S3). This highlights the potential of [^212^Pb]Pb-AB001 as a more effective treatment option for early metastases.

The disseminated model developed metastases in clinically relevant sites (Fig. 2, Table S1), replicating mCRPC tumour biology through IC inoculation, which drives metastases to bone and visceral tissues [35]. By contrast, previous studies using IV inoculation primarily resulted in pulmonary metastases (Table S4).

Selection of clinically relevant activities in this study was based on body surface area conversions [36]. With this approach, 0.24‒1.0 MBq [^212^Pb]Pb-AB001 correspond to human-equivalent activities of 45‒180 MBq, which align with the per-cycle activities of ^212^Pb evaluated in clinical trials [23, 24, 29, 37, 38]. Similarly, 43 MBq [^177^Lu]Lu-PSMA-617 approximates the clinically approved activity of 7.4 GBq per cycle [8, 36]. While selecting activities to achieve comparable toxicities or tumour absorbed doses could enable a more direct comparison, clinical toxicity data for [^212^Pb]Pb-AB001 is not yet available and there are challenges related to dosimetry of micrometastases. We previously reported a tumour absorbed dose of 11.1 Gy/MBq in PC-3 PIP tumours of athymic nude mice [27], translating to absorbed doses of 2.7, 5.6, and 11.1 Gy for the 0.24, 0.5, and 1 MBq activities used herein, respectively. For [^177^Lu]Lu-PSMA-617, the absorbed dose in a similar model was 3.9 ± 0.6 Gy/MBq [39–41], corresponding to 70‒99, 138‒194 and 211–297 Gy for the 22, 43 and 66 MBq activities used in this study, respectively. However, these estimates were obtained in subcutaneous tumours, where the absorbed fraction of both alpha and beta particles approach 100% [42]. In micrometastatic lesions (50–100 μm), the absorbed fraction of beta radiation is expected to be only around 10% [42, 43], resulting in absorbed doses of 7‒10, 14‒19, and 21–30 Gy for [^177^Lu]Lu-PSMA-617 herein. Although alpha particles also exhibit reduced absorbed fractions in small lesions, the effect is considerably less pronounced due to their short range and high LET [44, 45]. Additionally, the high LET of alpha particles results in significantly greater biological effectiveness per unit dose compared to beta particles [45]. Thus, [^212^Pb]Pb-AB001 may still produce greater biological effects than [^177^Lu]Lu-PSMA-617 despite a lower absorbed dose, which may explain the differences in therapeutic efficacy in this study. Nevertheless, absorbed doses of [^212^Pb]Pb-AB001 and [^177^Lu]Lu-PSMA-617 cannot be compared without accounting for dose rate and the relative biological effectiveness. Given the complexity of such calculations and limited clinical dosimetry data for [^212^Pb]Pb-AB001, the administered activities herein were based on clinically selected ^212^Pb activities to date [23, 24, 29, 37, 38], highlighting the need for further investigation to determine the generalisability of these findings.

Importantly, no toxicity was observed for either radioligand (Fig. S7). In addition to the stability of [^212^Pb]Pb-AB001 documented in serum and in a recent patient study [20, 30], this finding highlights [^212^Pb]Pb-AB001’s promise for clinical translation.

Increasing [^212^Pb]Pb-AB001 beyond 0.25 MBq offered a slight delay in metastatic progression (Fig. 5C‒D, Fig. S6 and Table S3), but no significant survival improvement (Fig. 5; Table 1). With approximately 0.25 × 10^6^ cells being treated with higher activities (2 MBq/µg), PSMA-binding sites on the cells may have become saturated. However, this is difficult to quantify because of PSMA expression in normal tissues, such as renal proximal tubules. Alternative strategies, like multiple low-activity injections have improved [^212^Pb]Pb-AB001 efficacy in subcutaneous PC-3 PIP xenografts [27], and evaluating this in metastatic models could also potentially yield valuable insights in future studies.

The limited efficacy of [^177^Lu]Lu-PSMA-617 observed in this study aligns with previous findings when administered ≤ 3 w post-cell inoculation (Table S4) [14, 25, 34, 46]. Antitumour efficacy has been reported only in millimetre-scale C4-2 metastases, reaching a TI of 1.3 when administered 5 w post-cell inoculation [14]. These observations underscore the importance of radionuclide properties, including particle pathlength and LET, for treating metastatic disease. Suboptimal responses of [^177^Lu]Lu-PSMA-617 to CTC (~ 7–25 μm) and smaller micrometastases (< 0.2 mm) suggest insufficient energy deposition from beta particles [11, 47–49]. Furthermore, given the identical PSMA-binding unit of AB001 and PSMA-617 [30], alongside comparable cell-bound activity and internalisation of [^212^Pb]Pb-AB001 and [^177^Lu]Lu-PSMA-617 (*p* < 0.05, Fig. 3), and similar reported tumour-uptake in subcutaneous PC-3 PIP xenografts [27, 50–52], the enhanced efficacy of [^212^Pb]Pb-AB001 is likely to be attributable predominantly to the physical properties of its alpha-emitting daughters rather than differences in ligand properties.

Unlike [^177^Lu]Lu-PSMA-617, PSMA-targeted alpha therapy has demonstrated efficacy when administered between 1 day and 5 w post-cell inoculation (Table S4) [14, 25, 34, 53–55]. Interestingly, 0.24‒1.0 MBq [^212^Pb]Pb-AB001 has achieved greater treatment efficacy than 3.7 MBq [^212^Pb]Pb-L2 and 1.5‒3.7 MBq [^211^At]At-3-Lu injected 1 and 7 d post-cell inoculation, respectively (Table S4) [25, 54]. Treatment with 0.37 MBq [^211^At]At-6, administered 1 day post-cell inoculation resulted in a TI of up to 1.9 [53]. Although [^212^Pb]Pb-AB001 efficacy under similar early post-inoculation conditions was not assessed herein, future studies on its impact on CTCs could be informative. ^225^Ac-PSMA-targeted alpha therapy has also shown promise preclinically and clinically, but concerns over salivary gland toxicity and availability limit its potential [12–15]. Nevertheless, it is noteworthy that [^225^Ac]Ac-PSMA-617 attained a TI of 1.8‒1.9 in the C4-2 model when given 3 and 5 w post IC inoculation, and a TI of 3.9 when administered 1 week post-cell inoculation [14]. Notably, control mice bearing C4-2 metastases (0.5 × 10⁶ cells, IC) had a median survival of 7–8.3 w [14, 15], compared to 3.6 w (25 d) for PC-3 PIP-luc (2.5 × 10⁶ cells, IC) in this study. This highlights different tumour aggressiveness and disease states at treatment onset, which can influence therapeutic strategies and decisions.

Despite the aggressiveness of the PC-3 PIP-luc model, metastatic lesions were too small for detection by µPET/µCT and SPECT/µCT at day 0. The limited spatial resolution of small-animal PET and SPECT scanners (~ 0.7–0.9 mm [56, 57]) prevents the detection of micrometastases < 0.7 mm. Additionally, ^212^Pb SPECT/µCT imaging is challenged by ^212^Pb decay chain properties [58], relatively short acquisition times (15–30 min), and discrepancies between activity levels required for imaging versus treatment, highlighting the need for optimised ^212^Pb SPECT/µCT protocols. Another limitation of this study is the use of PC-3 PIP-luc cells, which induce osteolytic rather than osteoblastic lesions typical of prostate cancer (Fig. 6A) [59, 60]. However, this study primarily aimed to evaluate the impact of targeted radioligand therapy on micrometastases rather than on mature bone lesions.

In conclusion, for the administered activities studied herein, [^212^Pb]Pb-AB001 demonstrated promising therapeutic efficacy in a preclinical model of disseminated prostate cancer, effectively delaying the development of visceral metastases and preventing formation of detectable bone metastases compared to [^177^Lu]Lu-PSMA-617. While the equivalence of the administered activities needs further study, these findings advocate for continued exploration and optimisation of [^212^Pb]Pb-AB001 in the clinical setting, particularly for patients at risk of early metastatic progression.

## Electronic supplementary material

Below is the link to the electronic supplementary material.

Supplementary Material 1

## Data Availability

All data generated and analysed during this study are included in this published article and its supplementary information files.

## References

[1] Kirby M, Hirst C, Crawford ED. Characterising the castration-resistant prostate cancer population: a systematic review. Int J Clin Pract. 2011;65:1180–92. 10.1111/j.1742-1241.2011.02799.x. 21995694 10.1111/j.1742-1241.2011.02799.x

[2] Nishino T, Yamamoto S, Numao N, Komai Y, Oguchi T, Yasuda Y, et al. Predictors of progression to Castration-resistant prostate Cancer after radical prostatectomy in High-risk prostate Cancer patients. Cancer Diagn Progn. 2024;4:646–51. 10.21873/cdp.10376. 39238616 10.21873/cdp.10376 PMC11372693

[3] Moreira DM, Howard LE, Sourbeer KN, Amarasekara HS, Chow LC, Cockrell DC, et al. Predictors of time to metastasis in Castration-resistant prostate Cancer. Urology. 2016;96:171–6. 10.1016/j.urology.2016.06.011. 27318265 10.1016/j.urology.2016.06.011 PMC5536963

[4] Mitchell AP, Meza AM, Panageas KS, Lipitz-Snyderman A, Farooki A, Morris MJ. Real-world use of bone modifying agents in metastatic, castration-resistant prostate cancer. Prostate Cancer Prostatic Dis. 2023;26:126–32. 10.1038/s41391-022-00573-y. 35798857 10.1038/s41391-022-00573-y PMC10251421

[5] Derlin T, Riethdorf S, Schumacher U, Lafos M, Peine S, Coith C, et al. PSMA-heterogeneity in metastatic castration-resistant prostate cancer: Circulating tumor cells, metastatic tumor burden, and response to targeted radioligand therapy. Prostate. 2023;83:1076–88. 10.1002/pros.24549. 37147881 10.1002/pros.24549

[6] Danila DC, Heller G, Gignac GA, Gonzalez-Espinoza R, Anand A, Tanaka E, et al. Circulating tumor cell number and prognosis in progressive castration-resistant prostate cancer. Clin Cancer Res. 2007;13:7053–8. 10.1158/1078-0432.Ccr-07-1506. 18056182 10.1158/1078-0432.CCR-07-1506

[7] Nakamura S, Nagata M, Nagaya N, Ashizawa T, Hirano H, Lu Y, et al. The detection and negative reversion of Circulating tumor cells as prognostic biomarkers for metastatic Castration-Resistant prostate Cancer with bone metastases treated by enzalutamide. Cancers (Basel). 2024;16. 10.3390/cancers16040772. 10.3390/cancers16040772 PMC10886552 38398163

[8] Sartor O, de Bono J, Chi KN, Fizazi K, Herrmann K, Rahbar K, et al. Lutetium-177-PSMA-617 for metastatic Castration-Resistant prostate Cancer. N Engl J Med. 2021;385:1091–103. 10.1056/NEJMoa2107322. 34161051 10.1056/NEJMoa2107322 PMC8446332

[9] ICRP. Nuclear Decay Data for Dosimetric Calculations. ICRP Publication 107. 2008. 10.1016/j.icrp.2008.10.004 19285593

[10] Schuchardt C, Zhang J, Kulkarni HR, Chen X, Müller D, Baum RP. Prostate-Specific membrane antigen radioligand therapy using (177)Lu-PSMA I&T and (177)Lu-PSMA-617 in patients with metastatic Castration-Resistant prostate cancer: comparison of safety, biodistribution, and dosimetry. J Nucl Med. 2022;63:1199–207. 10.2967/jnumed.121.262713. 34887335 10.2967/jnumed.121.262713 PMC9364353

[11] Miyazawa Y, Konik A, Otani K, Pittie R, Rodden D, Kelly J et al. Circulating tumor cell molecular signatures of response to 177Lu-PSMA-617 therapy in metastatic prostate cancer patients. J Nucl Med; 2024. p. 241193.

[12] Jalloul W, Ghizdovat V, Stolniceanu CR, Ionescu T, Grierosu IC, Pavaleanu I, et al. Targeted Alpha Therapy: All We Need to Know about (225)Ac’s Physical Characteristics and Production as a Potential Theranostic Radionuclide. Pharmaceuticals (Basel). 2023;16. 10.3390/ph16121679. 10.3390/ph16121679 PMC10747780 38139806

[13] Feuerecker B, Tauber R, Knorr K, Heck M, Beheshti A, Seidl C, et al. Activity and adverse events of Actinium-225-PSMA-617 in advanced metastatic Castration-resistant prostate Cancer after failure of Lutetium-177-PSMA. Eur Urol. 2021;79:343–50. 10.1016/j.eururo.2020.11.013. 33293081 10.1016/j.eururo.2020.11.013

[14] Meyer C, Stuparu A, Lueckerath K, Calais J, Czernin J, Slavik R, Dahlbom M. Tandem isotope therapy with (225)Ac- and (177)Lu-PSMA-617 in a murine model of prostate Cancer. J Nucl Med. 2023;64:1772–8. 10.2967/jnumed.123.265433. 37797974 10.2967/jnumed.123.265433 PMC10626377

[15] Stuparu AD, Meyer CAL, Evans-Axelsson SL, Lückerath K, Wei LH, Kim W, et al. Targeted alpha therapy in a systemic mouse model of prostate cancer - a feasibility study. Theranostics. 2020;10:2612–20. 10.7150/thno.42228. 32194823 10.7150/thno.42228 PMC7052903

[16] Zimmermann R. Is (212)Pb really happening?? The Post-(177)Lu/(225)Ac blockbuster?? J Nucl Med. 2024;65:176–7. 10.2967/jnumed.123.266774. 38176723 10.2967/jnumed.123.266774

[17] Kokov KV, Egorova BV, German MN, Klabukov ID, Krasheninnikov ME, Larkin-Kondrov AA, et al. (212)Pb: production approaches and targeted therapy applications. Pharmaceutics. 2022;14. 10.3390/pharmaceutics14010189. 10.3390/pharmaceutics14010189 PMC8777968 35057083

[18] Kvassheim M, Revheim MR, Stokke C. Quantitative SPECT/CT imaging of lead-212: a Phantom study. EJNMMI Phys. 2022;9:52. 10.1186/s40658-022-00481-z. 35925521 10.1186/s40658-022-00481-z PMC9352840

[19] Berner K, Hernes E, Kvassheim M, Revheim M-E, Bastiansen J, Selboe S et al. First-in-human study of AB001, a prostate-specific membrane antigen (PSMA) targeted 212Pb alpha radioligand, in patients with metastatic castration resistant prostate cancer (mCRPC): Phase 0 experience. EMUC24–16th European Multidisciplinary Congress on Urological Cancers. Lisbon, Portugal; 2024.

[20] Berner K, Hernes E, Kvassheim M, Revheim ME, Bastiansen J, Selboe S, et al. First-in-Human phase 0 study of AB001, a prostate-Specific membrane Antigen-Targeted (212)Pb Radioligand, in patients with metastatic Castration-Resistant prostate Cancer. J Nucl Med. 2025. 10.2967/jnumed.124.269299. 40081958 10.2967/jnumed.124.269299 PMC12051763

[21] Griffiths MR, Pattison DA, Latter M, Kuan K, Taylor S, Tieu W, et al. First-in-Human 212Pb-PSMA–Targeted α-Therapy SPECT/CT imaging in a patient with metastatic Castration-Resistant prostate Cancer. J Nucl Med. 2024;65:664. 10.2967/jnumed.123.267189. 38423783 10.2967/jnumed.123.267189 PMC10995529

[22] Kvassheim M, Tornes AJK, Juzeniene A, Stokke C, Revheim MR. Imaging of (212)Pb in mice with a clinical SPECT/CT. EJNMMI Phys. 2023;10:47. 10.1186/s40658-023-00571-6. 37603123 10.1186/s40658-023-00571-6 PMC10442031

[23] Delpassand ES, Tworowska I, Esfandiari R, Torgue J, Hurt J, Shafie A, Núñez R. Targeted α-Emitter therapy with (212)Pb-DOTAMTATE for the treatment of metastatic SSTR-Expressing neuroendocrine tumors: First-in-Humans Dose-Escalation clinical trial. J Nucl Med. 2022;63:1326–33. 10.2967/jnumed.121.263230. 34992153 10.2967/jnumed.121.263230 PMC9454455

[24] Meredith RF, Torgue JJ, Rozgaja TA, Banaga EP, Bunch PW, Alvarez RD, et al. Safety and outcome measures of First-in-Human intraperitoneal α radioimmunotherapy with 212Pb-TCMC-Trastuzumab. Am J Clin Oncol. 2018;41:716–21. 10.1097/coc.0000000000000353. 27906723 10.1097/COC.0000000000000353 PMC5449266

[25] Banerjee SR, Minn I, Kumar V, Josefsson A, Lisok A, Brummet M, et al. Preclinical evaluation of (203/212)Pb-Labeled Low-Molecular-Weight compounds for targeted radiopharmaceutical therapy of prostate Cancer. J Nucl Med. 2020;61:80–8. 10.2967/jnumed.119.229393. 31253744 10.2967/jnumed.119.229393 PMC6954458

[26] Stenberg VY, Larsen RH, Ma LW, Peng Q, Juzenas P, Bruland ØS, Juzeniene A. Evaluation of the PSMA-Binding ligand (212)Pb-NG001 in multicellular tumour spheroid and mouse models of prostate Cancer. Int J Mol Sci. 2021;22. 10.3390/ijms22094815. 10.3390/ijms22094815 PMC8124365 34062920

[27] Stenberg VY, Tornes AJK, Nilsen HR, Revheim ME, Bruland ØS, Larsen RH, Juzeniene A. Factors influencing the therapeutic efficacy of the PSMA targeting radioligand (212)Pb-NG001. Cancers (Basel). 2022;14. 10.3390/cancers14112784. 10.3390/cancers14112784 PMC9179904 35681766

[28] Li J, Huang T, Hua J, Wang Q, Su Y, Chen P, et al. CD46 targeted 212Pb alpha particle radioimmunotherapy for prostate cancer treatment. J Experimental Clin Cancer Res. 2023;42:61. 10.1186/s13046-023-02636-x. 10.1186/s13046-023-02636-x PMC10007843 36906664

[29] Strosberg JR, Naqvi S, Cohn AL, Delpassand E, Wagner VJ, Torgue J, et al. Safety, tolerability and efficacy of Pb-DOTAMTATE as a targeted alpha therapy for subjects with unresectable or metastatic somatostatin receptor-expressing gastroenteropancreatic neuroendocrine tumors (SSTR plus GEP-NETs): A phase 2 study. Journal of Clinical Oncology; 2024.

[30] Stenberg VY, Juzeniene A, Chen Q, Yang X, Bruland ØS, Larsen RH. Preparation of the alpha-emitting prostate-specific membrane antigen targeted radioligand [(212) Pb]Pb-NG001 for prostate cancer. J Label Comp Radiopharm. 2020;63:129–43. 10.1002/jlcr.3825. 10.1002/jlcr.3825 31919866

[31] Larsen RH. Production of highly purified 212Pb. 2010.

[32] Li RG, Stenberg VY, Larsen RH. An experimental generator for production of High-Purity (212)Pb for use in radiopharmaceuticals. J Nucl Med. 2023;64:173–6. 10.2967/jnumed.122.264009. 35798556 10.2967/jnumed.122.264009 PMC9841245

[33] Stenberg VY, Juzeniene A, Bruland ØS, Larsen RH. In situ generated (212)Pb-PSMA ligand in a (224)Ra-Solution for dual targeting of prostate Cancer sclerotic stroma and PSMA-positive cells. Curr Radiopharm. 2020;13:130–41. 10.2174/1874471013666200511000532. 32389119 10.2174/1874471013666200511000532 PMC7527546

[34] Lückerath K, Bailis J, Current K, Salvati M, Radu C, Czernin J. 717 AMG 160, a prostate-specific membrane antigen (PSMA)-targeted BiTE^®^ immuno-oncology therapy, is active in models of advanced prostate cancer that are resistant to radioligand therapy. J Immunother Cancer. 2020;8:A429–30. 10.1136/jitc-2020-SITC2020.0717.

[35] Labitzky V, Baranowsky A, Maar H, Hanika S, Starzonek S, Ahlers AK, et al. Modeling spontaneous bone metastasis formation of solid human tumor xenografts in mice. Cancers (Basel). 2020;12. 10.3390/cancers12020385. 10.3390/cancers12020385 PMC7072706 32046143

[36] Nair AB, Jacob S. A simple practice guide for dose conversion between animals and human. J Basic Clin Pharm. 2016;7:27–31. 10.4103/0976-0105.177703. 27057123 10.4103/0976-0105.177703 PMC4804402

[37] Prasad V, Trikalinos N, Hanna A, Johnson F, Puhlmann M, Wahl R. A phase I/IIa of [212Pb]VMT-NET targeted Alpha-Particle therapy for advanced SSTR2 positive neuroendocrine tumors. J Nucl Med. 2024;65.

[38] Hansen AR, Pattison DA, Ngai S, Karmann A, Walker AJ, Campbell L, et al. Phase Ib/IIa dose escalation and expansion study of [< sup > 212 Pb]Pb-ADVC001 in metastatic castration-resistant prostate cancer: TheraPb–phase I/II study. Journal of Clinical Oncology. 2025;43:TPS275-TPS. doi:10.1200/JCO.2025.43.5_suppl.TPS275.

[39] Tschan VJ, Borgna F, Busslinger SD, Stirn M, Rodriguez JMM, Bernhardt P, et al. Preclinical investigations using [(177)Lu]Lu-Ibu-DAB-PSMA toward its clinical translation for radioligand therapy of prostate cancer. Eur J Nucl Med Mol Imaging. 2022;49:3639–50. 10.1007/s00259-022-05837-2. 35635566 10.1007/s00259-022-05837-2 PMC9399046

[40] Müller C, Umbricht CA, Gracheva N, Tschan VJ, Pellegrini G, Bernhardt P, et al. Terbium-161 for PSMA-targeted radionuclide therapy of prostate cancer. Eur J Nucl Med Mol Imaging. 2019;46:1919–30. 10.1007/s00259-019-04345-0. 31134301 10.1007/s00259-019-04345-0 PMC6820371

[41] Umbricht CA, Köster U, Bernhardt P, Gracheva N, Johnston K, Schibli R, et al. Alpha-PET for prostate cancer: preclinical investigation using (149)Tb-PSMA-617. Sci Rep. 2019;9:17800. 10.1038/s41598-019-54150-w. 31780798 10.1038/s41598-019-54150-w PMC6882876

[42] Hindié E, Zanotti-Fregonara P, Quinto MA, Morgat C, Champion C. Dose deposits from 90Y, 177Lu, 111In, and 161 tb in micrometastases of various sizes: implications for radiopharmaceutical therapy. J Nucl Med. 2016;57:759–64. 10.2967/jnumed.115.170423. 26912441 10.2967/jnumed.115.170423

[43] Goddu SM, Rao DV, Howell RW. Multicellular dosimetry for micrometastases: dependence of self-dose versus cross-dose to cell nuclei on type and energy of radiation and subcellular distribution of radionuclides. J Nucl Med. 1994;35:521–30. 8113908

[44] Feinendegen LE, McClure JJ. Alpha-Emitters for medical therapy: workshop of the united States department of energy: Denver, Colorado, May 30–31, 1996. Radiat Res. 1997;148. 10.2307/3579579.

[45] Sgouros G, Roeske JC, McDevitt MR, Palm S, Allen BJ, Fisher DR, et al. MIRD pamphlet 22 (abridged): radiobiology and dosimetry of alpha-particle emitters for targeted radionuclide therapy. J Nucl Med. 2010;51:311–28. 10.2967/jnumed.108.058651. 20080889 10.2967/jnumed.108.058651 PMC5680544

[46] Stuparu AD, Capri JR, Meyer CAL, Le TM, Evans-Axelsson SL, Current K, et al. Mechanisms of resistance to prostate-Specific membrane Antigen–Targeted radioligand therapy in a mouse model of prostate Cancer. J Nucl Med. 2021;62:989–95. 10.2967/jnumed.120.256263. 33277393 10.2967/jnumed.120.256263 PMC8882874

[47] Chen JF, Ho H, Lichterman J, Lu YT, Zhang Y, Garcia MA, et al. Subclassification of prostate cancer Circulating tumor cells by nuclear size reveals very small nuclear Circulating tumor cells in patients with visceral metastases. Cancer. 2015;121:3240–51. 10.1002/cncr.29455. 25975562 10.1002/cncr.29455 PMC4560974

[48] Ribeiro-Samy S, Oliveira MI, Pereira-Veiga T, Muinelo-Romay L, Carvalho S, Gaspar J, et al. Fast and efficient microfluidic cell filter for isolation of Circulating tumor cells from unprocessed whole blood of colorectal cancer patients. Sci Rep. 2019;9:8032. 10.1038/s41598-019-44401-1. 31142796 10.1038/s41598-019-44401-1 PMC6541613

[49] Coumans FA, van Dalum G, Beck M, Terstappen LW. Filter characteristics influencing Circulating tumor cell enrichment from whole blood. PLoS ONE. 2013;8:e61770. 10.1371/journal.pone.0061770. 23626725 10.1371/journal.pone.0061770 PMC3634026

[50] Benešová M, Umbricht CA, Schibli R, Müller C, Albumin-Binding PSMA, Ligands. Optimization of the tissue distribution profile. Mol Pharm. 2018;15:934–46. 10.1021/acs.molpharmaceut.7b00877. 29400475 10.1021/acs.molpharmaceut.7b00877

[51] Tschan VJ, Borgna F, Schibli R, Müller C. Impact of the mouse model and molar amount of injected ligand on the tissue distribution profile of PSMA radioligands. Eur J Nucl Med Mol Imaging. 2021. 10.1007/s00259-021-05446-5. 34402925 10.1007/s00259-021-05446-5 PMC8803738

[52] Umbricht CA, Benešová M, Schmid RM, Türler A, Schibli R, van der Meulen NP, Müller C. (44)Sc-PSMA-617 for radiotheragnostics in tandem with (177)Lu-PSMA-617-preclinical investigations in comparison with (68)Ga-PSMA-11 and (68)Ga-PSMA-617. EJNMMI Res. 2017;7:9. 10.1186/s13550-017-0257-4. 28102507 10.1186/s13550-017-0257-4 PMC5247395

[53] Kiess AP, Minn I, Vaidyanathan G, Hobbs RF, Josefsson A, Shen C, et al. (2S)-2-(3-(1-Carboxy-5-(4-211At-Astatobenzamido)Pentyl)Ureido)-Pentanedioic acid for PSMA-Targeted α-Particle radiopharmaceutical therapy. J Nucl Med. 2016;57:1569–75. 10.2967/jnumed.116.174300. 27230930 10.2967/jnumed.116.174300 PMC5367442

[54] Mease RC, Kang CM, Kumar V, Banerjee SR, Minn I, Brummet M, et al. An improved (211)At-Labeled agent for PSMA-Targeted α-Therapy. J Nucl Med. 2022;63:259–67. 10.2967/jnumed.121.262098. 34088772 10.2967/jnumed.121.262098 PMC8805774

[55] Banerjee SR, Lisok A, Minn I, Josefsson A, Kumar V, Brummet M, et al. Preclinical evaluation of (213)Bi- and (225)Ac-Labeled Low-Molecular-Weight compounds for radiopharmaceutical therapy of prostate Cancer. J Nucl Med. 2021;62:980–8. 10.2967/jnumed.120.256388. 33246975 10.2967/jnumed.120.256388 PMC8882883

[56] Adler SS, Seidel J, Choyke PL. Advances in preclinical PET. Semin Nucl Med. 2022;52:382–402. 10.1053/j.semnuclmed.2022.02.002. 35307164 10.1053/j.semnuclmed.2022.02.002 PMC9038721

[57] Amirrashedi M, Zaidi H, Ay MR. Advances in preclinical PET instrumentation. PET Clin. 2020;15:403–26. 10.1016/j.cpet.2020.06.003. 32768368 10.1016/j.cpet.2020.06.003

[58] Mikalsen LTG, Kvassheim M, Stokke C. Optimized SPECT imaging of (224)Ra α-Particle therapy by (212)Pb photon emissions. J Nucl Med. 2023;64:1131–7. 10.2967/jnumed.122.264455. 37268424 10.2967/jnumed.122.264455 PMC10315694

[59] Ibrahim T, Flamini E, Mercatali L, Sacanna E, Serra P, Amadori D. Pathogenesis of osteoblastic bone metastases from prostate cancer. Cancer. 2010;116:1406–18. 10.1002/cncr.24896. 20108337 10.1002/cncr.24896

[60] Yin JJ, Pollock CB, Kelly K. Mechanisms of cancer metastasis to the bone. Cell Res. 2005;15:57–62. 10.1038/sj.cr.7290266. 15686629 10.1038/sj.cr.7290266

