# Supplementary material for: Therapeutic evaluation of [212Pb]Pb-AB001 and [177Lu]Lu-PSMA-617 in a mouse model of disseminated prostate cancer
Source: Eur J Nucl Med Mol Imaging. 2025 May 21;52(13):4847–59. doi: 10.1007/s00259-025-07330-y (PMC12589369; doi:10.1007/s00259-025-07330-y)
Supplement: Supplementary file 1 — Supplementary Material 1 [file 259_2025_7330_MOESM1_ESM.docx]

**Supplementary Information**

**Therapeutic evaluation of [^212^Pb]Pb-AB001 and [^177^Lu]Lu-PSMA-617 in a mouse model of disseminated prostate cancer**

Anna Julie Kjøl Høyvik^1,2,3^, Monika Kvassheim^3,4^, Li-Wei Ma^1^, Elisabeth Wiig^1^, Tiril Hillestad^5^, Mona-Elisabeth Revheim^3,6^, Rugile Liukaityte^1,2^, Øyvind Bruland^3,7^ and Asta Juzeniene^1,8,*^

1. Department of Radiation Biology, Institute for Cancer Research, Oslo University Hospital, 0379 Oslo, Norway
2. ARTBIO AS, 0379 Oslo, Norway
3. Faculty of Medicine, Institute of Clinical Medicine, University of Oslo, 0318 Oslo, Norway
4. Department of Physics and Computational Radiology, Institute for Cancer Research and Molecular Imaging, Oslo University Hospital, 0379 Oslo, Norway
5. Department of Core Facilities, Institute for Cancer Research and Molecular Imaging, Oslo University Hospital, 0379 Oslo, Norway
6. The Intervention Centre, Oslo University Hospital, 0372 Oslo, Norway
7. Department of Oncology, Institute for Cancer Research, Oslo University Hospital, 0379 Oslo, Norway
8. Faculty of Physics, University of Oslo, 0318 Oslo, Norway

* Correspondence: astaj@ous-hf.no; Tel.: +47 998 74 871

**Supplementary Methods**

**Transduction of cells**


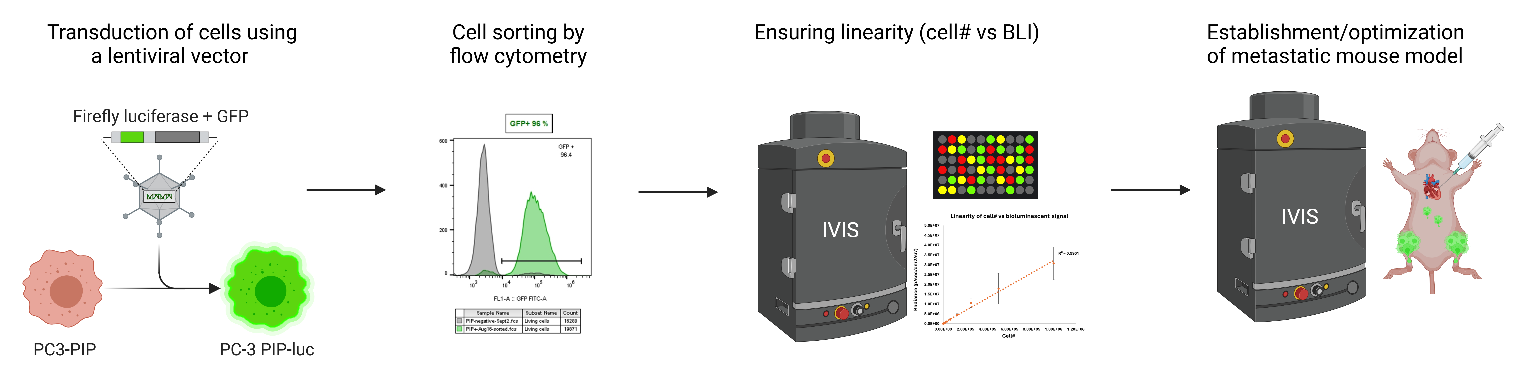


**Figure S1.** **Development and optimisation of the metastatic PC-3 PIP-luc mouse model.** The cells were transduced with RediFect Red-FLuc-GFP Lentiviral Particles followed by fluorescence activated cell sorting to isolate successfully transduced cells. The resulting batch was referred to as PC-3 PIP-luc. Linearity between cell number and bioluminescent signal was confirmed by bioluminescence imaging using an IVIS Spectrum Xenogen instrument (Figure S3). The mouse model was established and optimised by injecting varying numbers of PC-3 PIP-luc cells intracardially and monitoring the formation of metastases by bioluminescence imaging. The figure was created in BioRender.

**Therapeutic efficacy**

Mice were monitored for changes in body weight (2‒3 times per week), metastatic burden by 2D BLI (once per week) and humane endpoint criteria including >20% weight loss from initial body weight, rapid weight loss of >10%, maximum BLI intensity exceeding 10^8^ p/s/cm^2^/sr, ascites, paraplegia or any signs of severe sickness or discomfort. At the humane or experimental endpoint, blood was collected from the saphenous vein and complete blood counts were obtained by using the hematology analyser scil Vet abs (Kruuse, Drøbak, Norway). Blood was also drawn by cardiac puncture under gas anaesthesia, from which serum was collected and analysed using the Reflotron Plus (Roche Diagnostics AS, Oslo, Norway) or InSight V-CHEM Veterinary Chemistry Analyser (Lifetest Vet Equipment, Rødovre, Denmark). Selected organs (liver, spleen, kidneys, salivary glands, femurs and skull) were harvested and weighed.


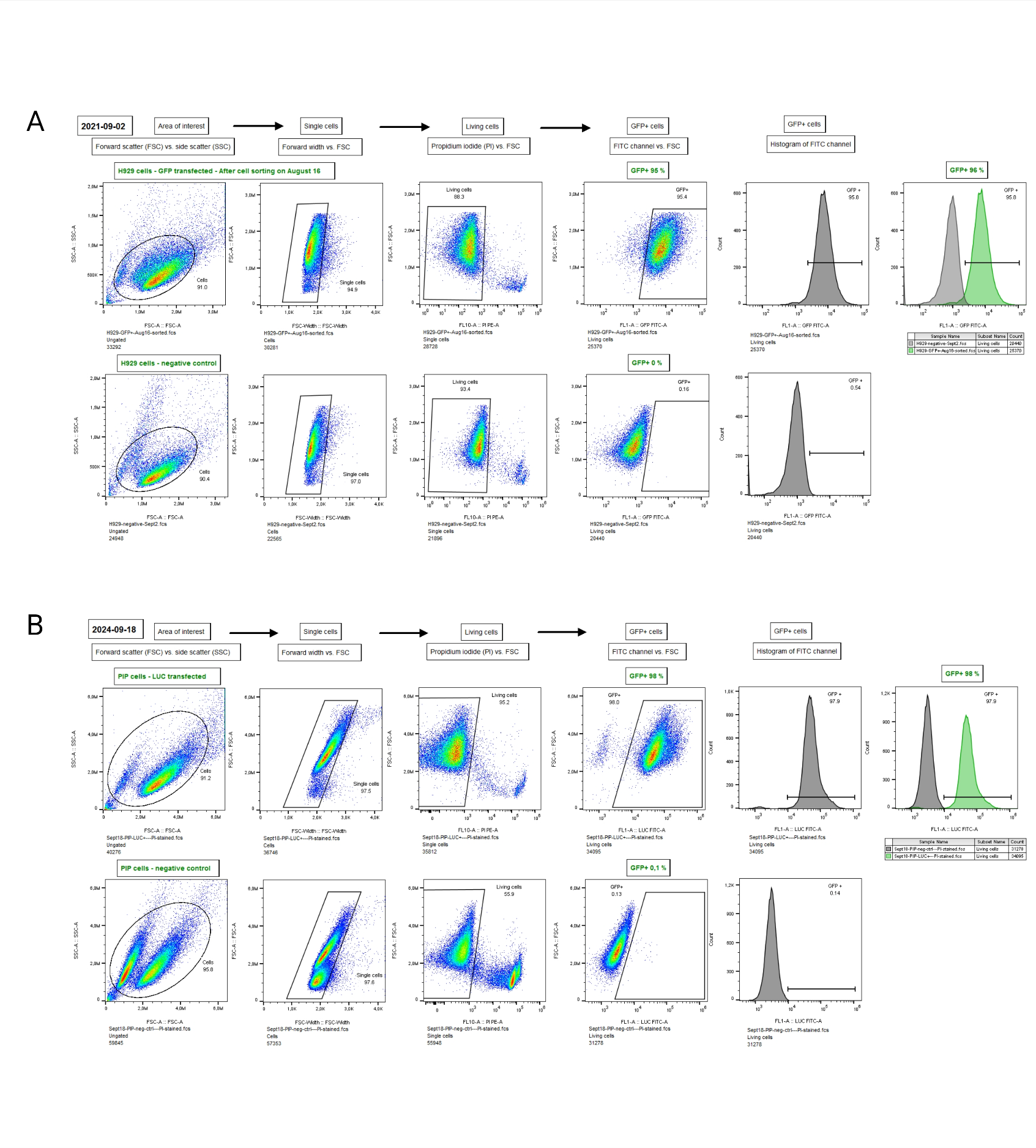
**Supplementary Results**

**Figure S2.** **Stability of GFP expression in PC-3 PIP-luc cells over time.** Detection of GFP-expressing PC-3 PIP-luc cells by flow cytometry (A) in 2021, three weeks post transfection and sorting of cells from non-transduced PC-3 PIP cells to ensure stable initial transfection of cells, and (B) in 2024 after the final animal studies presented in this paper to confirm the persistence of transfection. This analysis demonstrates consistent and stable expression of the lentiviral vector in the PC-3 PIP-luc cells throughout the study period.


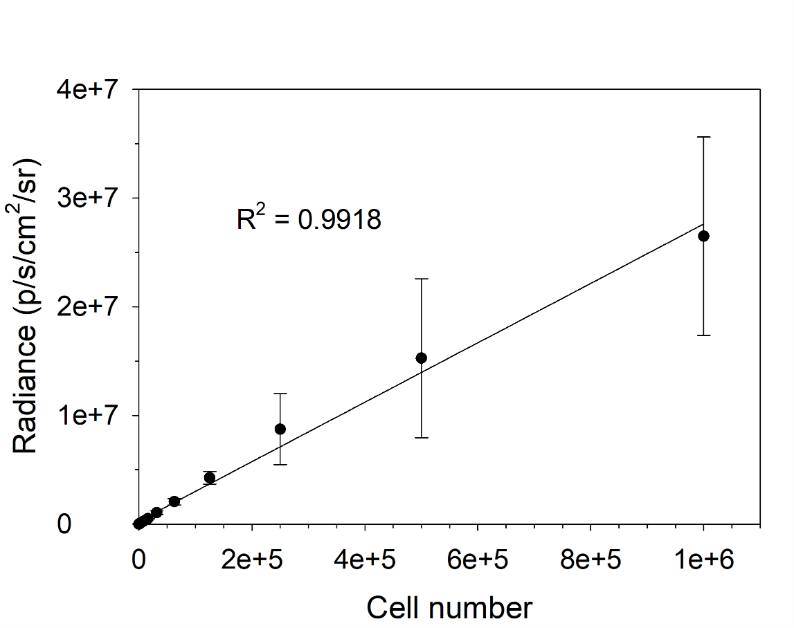


**Figure S3.** **Correlation between PC-3 PIP-luc cell count and bioluminescent signal intensity (photons/sec/cm²/sr).** Data represents the mean values from three independent experiments (N=3), demonstrating a strong linear correlation between cell quantity and bioluminescent signal intensity.

**
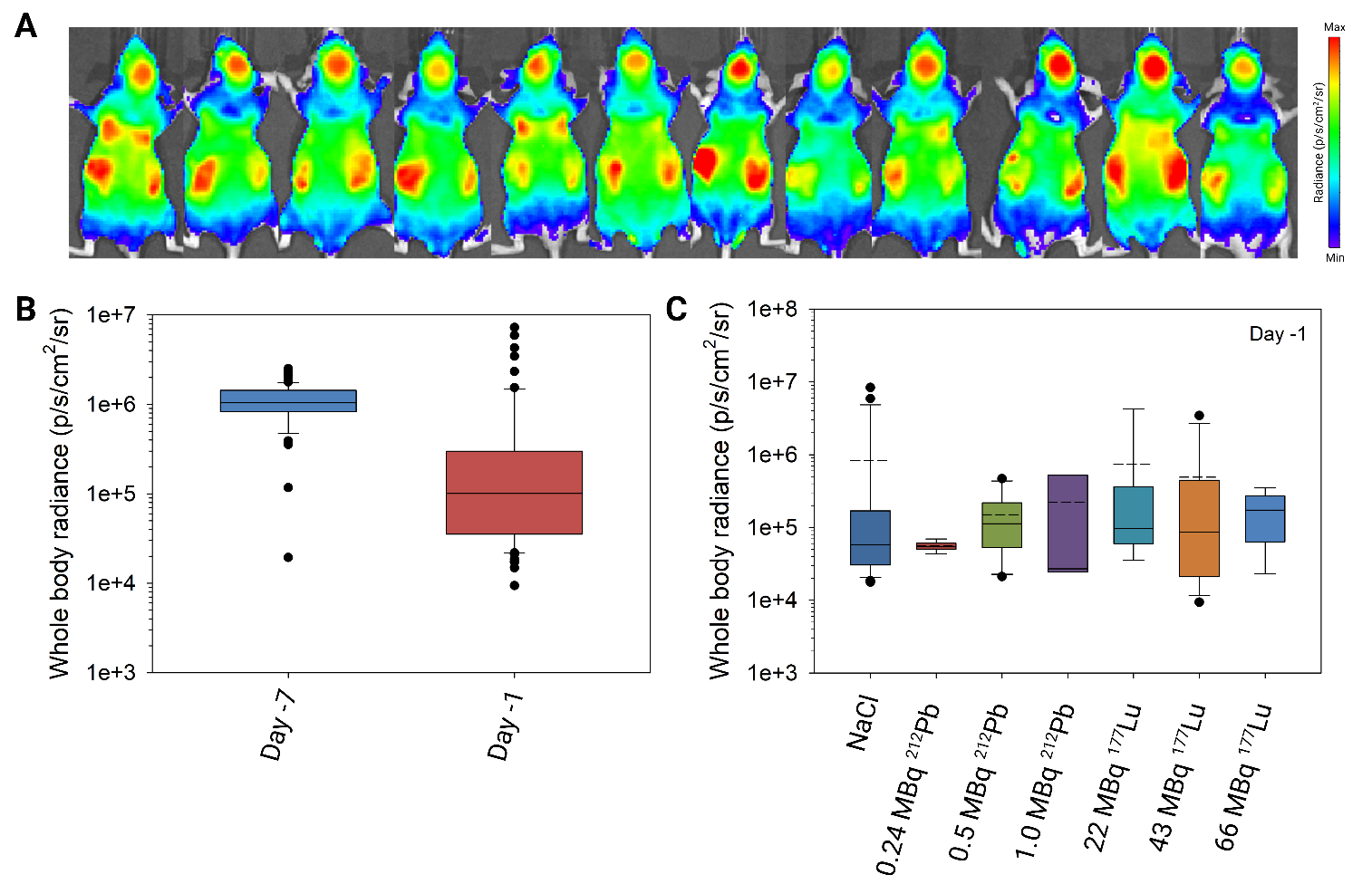
**

**Figure S4.** (A) Representative 2D bioluminescence images of mice taken immediately post intracardiac cell inoculation (day -7), illustrating the initial dissemination and distribution of PC-3 PIP-luc cells throughout the cardiovascular system. (B) Whole body bioluminescence radiance plots (photons/sec/cm²/sr) of all mice included in the study at the cell inoculation day (day -7) and at the day before treatment (day -1). (C) Whole body bioluminescence radiance plots (photons/sec/cm²/sr) of the control group (NaCl) and the groups treated with 0.24, 0.5 or 1.0 MBq [^212^Pb]Pb-AB001 or 22, 43 or 66 MBq [^177^Lu]Lu-PSMA-617 at the day before treatment. The bottom of the boxes represents the 25th percentiles, solid lines represent the medians, dashed lines represent the mean, the top of the boxes represent the 75th percentiles and whiskers represent the 5th and 95th percentiles.


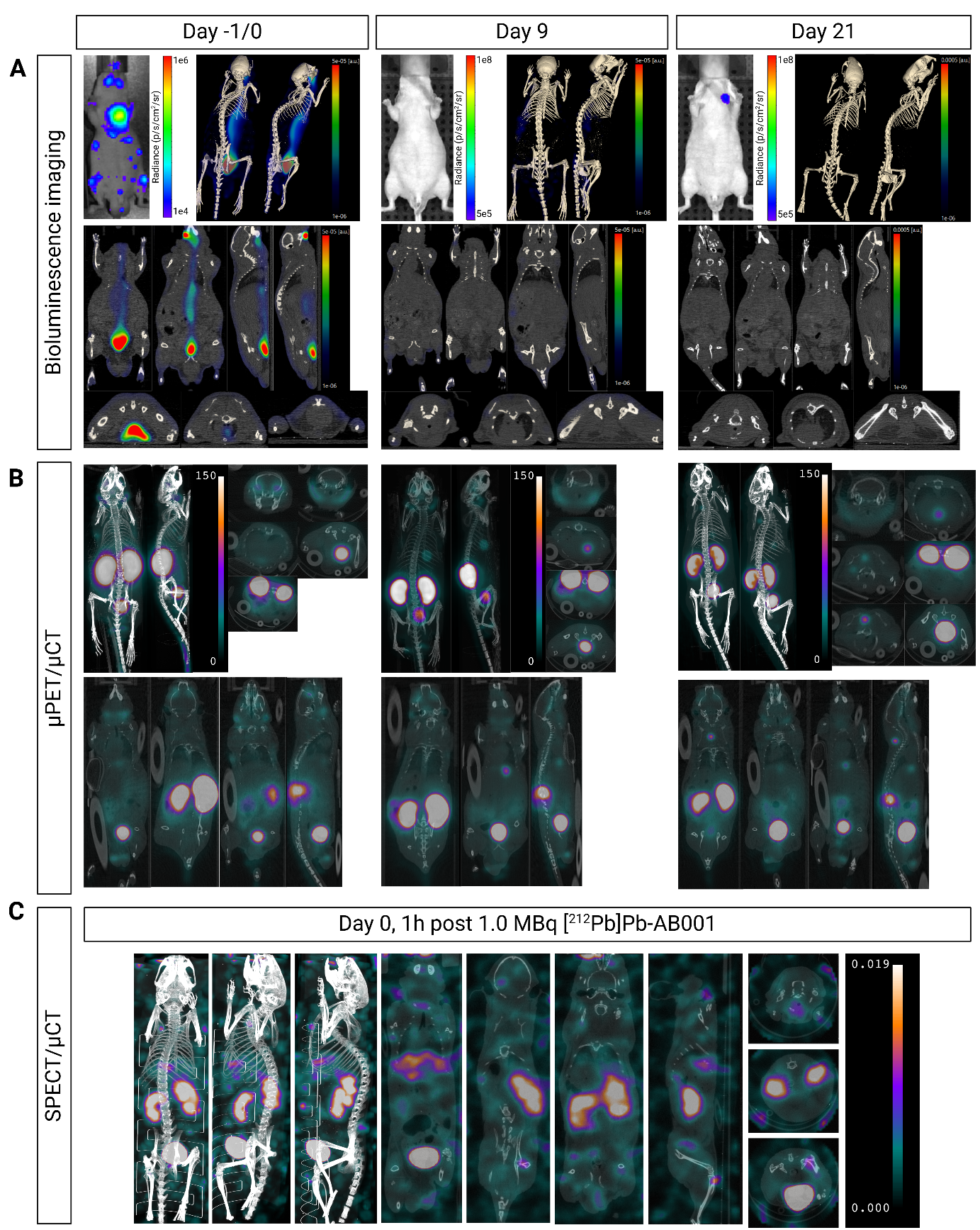


**Figure S5.** **Representative images of a mouse** **intracardially (IC) injected with 2.5 × 10^6^ PC-3 PIP-luc cells on day -7 and treated with 1.0 MBq [^212^Pb]Pb-AB001 on day 0.** (A) BLI images were captured on day -1 (one day before therapy), and continued weekly until day 21, providing longitudinal insights into tumour progression and metastatic spread post treatment. (B) µPET/µCT imaging was performed 2 h post intravenous administration of 5 MBq [^18^F]F-PSMA-1007 on day 0, 9 and 21, highlighting PSMA-expression and uptake of the imaging radioligand. (C) SPECT/CT imaging was performed on day 0, 1 h post intravenous administration of 1.0 MBq [^212^Pb]Pb-AB001, to identify its biodistribution.

**Table S1.** **Tissue-specific metastatic incidence in control mice at the humane endpoint.** Nine organs from seven control mice were harvested at the humane endpoint. The imaging was conducted using the IVIS Xenogen system to identify the presence of tissue-specific metastases in the model.

| **Organ** | **Number of mice** | **Incidence (%)** |
| --- | --- | --- |
| Lung | 5/7 | 71 % |
| Liver | 4/7 | 57 % |
| Femur(s) | 7/7 | 100 % |
| Skull | 4/7 | 57 % |
| Salivary glands | 3/7 | 43 % |
| Brain | 3/7 | 43 % |
| Heart | 3/7 | 43 % |
| Kidney | 2/7 | 29 % |
| Spleen | 0 | 0 % |

**Table S2.** Statistical analysis of survival among treatment groups. The statistical significance was estimated by log-rank test with multiple pairwise comparisons (Holm-Sidak). Statistical significant values are marked in green, while non-significant values are shown in red.

| **Comparison** | **P-value** |
| --- | --- |
| NaCl vs. 0.25 MBq [^212^Pb]Pb-AB001 | 0.002 |
| NaCl vs. 0.5 MBq [^212^Pb]Pb-AB001 | <0.001 |
| NaCl vs. 1.0 MBq [^212^Pb]Pb-AB001 | 0.001 |
| NaCl vs. 22 MBq [^177^Lu]Lu-PSMA-617 | 0.132 |
| NaCl vs. 43 MBq [^177^Lu]Lu-PSMA-617 | 0.930 |
| NaCl vs. 66 MBq [^177^Lu]Lu-PSMA-617 | 0.930 |
| 0.24 MBq [^212^Pb]Pb-AB001 vs. 22 MBq [^177^Lu]Lu-PSMA-617 | 0.004 |
| 0.24 MBq [^212^Pb]Pb-AB001 vs. 43 MBq [^177^Lu]Lu-PSMA-617 | 0.008 |
| 0.24 MBq [^212^Pb]Pb-AB001 vs. 66 MBq [^177^Lu]Lu-PSMA-617 | 0.035 |
| 0.5 MBq [^212^Pb]Pb-AB001 vs. 22 MBq [^177^Lu]Lu-PSMA-617 | <0.001 |
| 0.5 MBq [^212^Pb]Pb-AB001 vs. 43 MBq [^177^Lu]Lu-PSMA-617 | <0.001 |
| 0.5 MBq [^212^Pb]Pb-AB001 vs. 66 MBq [^177^Lu]Lu-PSMA-617 | 0.003 |
| 1.0 MBq [^212^Pb]Pb-AB001 vs. 22 MBq [^177^Lu]Lu-PSMA-617 | 0.008 |
| 1.0 MBq [^212^Pb]Pb-AB001 vs. 44 MBq [^177^Lu]Lu-PSMA-617 | 0.004 |
| 1.0 MBq [^212^Pb]Pb-AB001 vs. 66 MBq [^177^Lu]Lu-PSMA-617 | 0.008 |
| 0.25 MBq [^212^Pb]Pb-AB001 vs. 0.5 MBq [^212^Pb]Pb-AB001 | 0.930 |
| 0.25 MBq [^212^Pb]Pb-AB001 vs. 1.0 MBq [^212^Pb]Pb-AB001 | 0.930 |
| 0.5 MBq [^212^Pb]Pb-AB001 vs. 1.0 MBq [^212^Pb]Pb-AB001 | 0.930 |
| 22 MBq [^177^Lu]Lu-PSMA-617 vs. 43 MBq [^177^Lu]Lu-PSMA-617 | 0.010 |
| 22 MBq [^177^Lu]Lu-PSMA-617 vs. 66 MBq [^177^Lu]Lu-PSMA-617 | 0.020 |
| 43 MBq [^177^Lu]Lu-PSMA-617 vs. 66 MBq [^177^Lu]Lu-PSMA-617 | 0.951 |

**
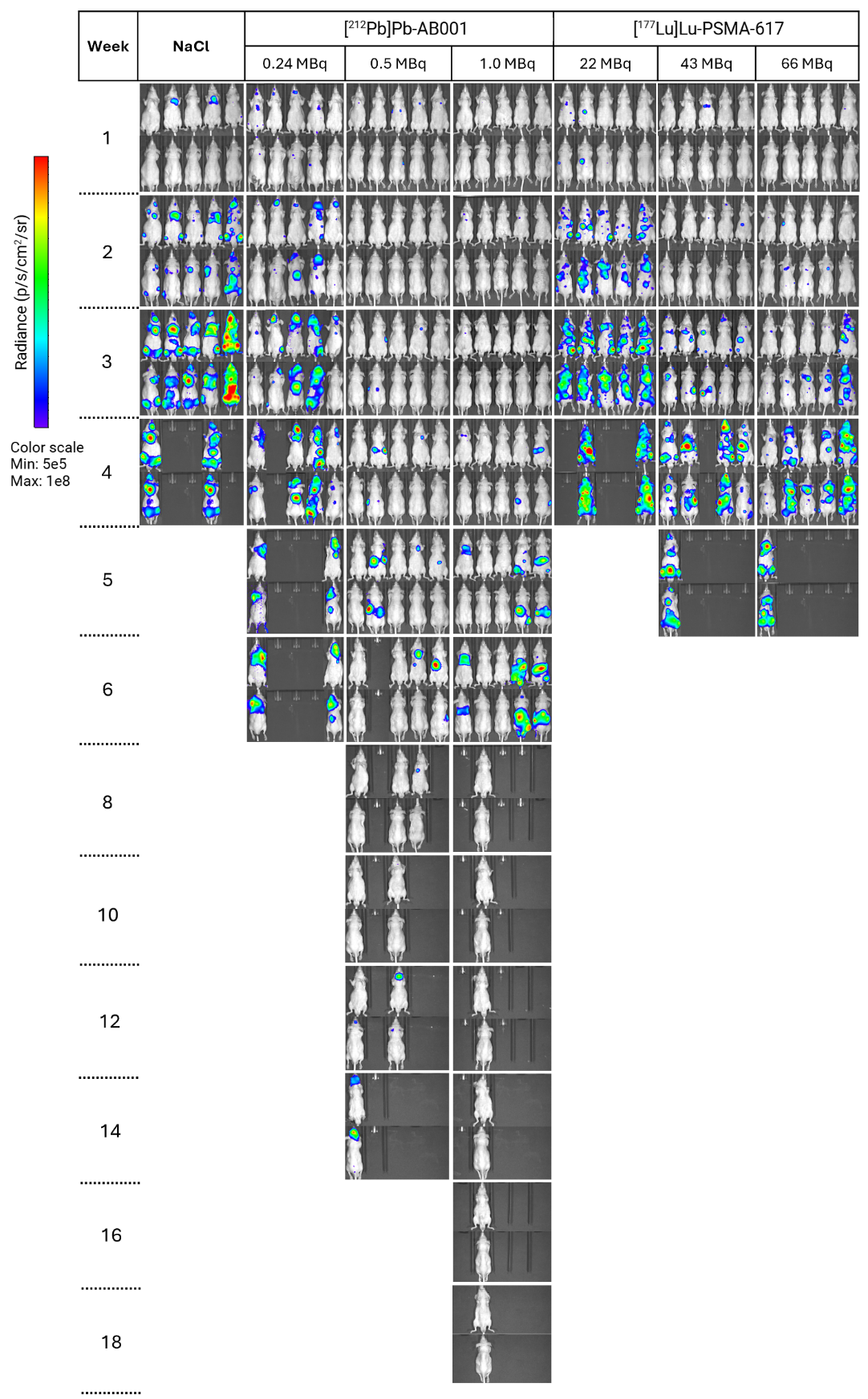
**

**Figure S6.** Representative 2D bioluminescence images of disseminated xenografts demonstrating metastatic burden over time, from one week after treatment with NaCl, [^212^Pb]Pb-AB001 or [^177^Lu]Lu-PSMA-617 until humane or experimental endpoint, with colour-mapped radiance values (photons/sec/cm²/sr).

**Table S3**. P-values from multiple pairwise comparisons of metastatic burden between groups (One Way ANOVA). Green colour indicates p-value < 0.05, red colour indicates p-value > 0.05 and grey colour indicates that one or several mice in one of the treated groups had been sacrificed at this time point.

|  | **P-value** | | | | |
| --- | --- | --- | --- | --- | --- |
| **Comparison** | **Week 0** | **Week 1** | **Week 2** | **Week 3** | **Week 4** |
| NaCl vs. 0.24 MBq ^212^Pb | *  0.616 | 0.071 | 0.005 | 0.777 |  |
| NaCl vs. 0.5 MBq ^212^Pb |  | 0.207 | < 0.001 | < 0.001 |  |
| NaCl vs. 1.0 MBq ^212^Pb |  | 0.163 | < 0.001 | 0.002 |  |
| NaCl vs. 22 MBq ^177^Lu |  | 1 | 1 | 1 |  |
| NaCl vs. 43 MBq ^177^Lu |  | 0.003 | 0.006 | 1 |  |
| NaCl vs. 66 MBq ^177^Lu |  | 0.019 | 0.033 | 1 |  |
| 0.24 MBq ^212^Pb vs. 22 MBq ^177^Lu |  | 1 | 0.041 | 0.326 |  |
| 0.24 MBq ^212^Pb vs. 43 MBq ^177^Lu |  | 1 | 1 | 1 |  |
| 0.24 MBq ^212^Pb vs. 66 MBq ^177^Lu |  | 1 | 1 | 1 |  |
| 0.5 MBq ^212^Pb vs. 22 MBq ^177^Lu |  | 1 | < 0.001 | < 0.001 |  |
| 0.5 MBq ^212^Pb vs. 43 MBq ^177^Lu |  | 1 | 0.331 | 0.004 |  |
| 0.5 MBq ^212^Pb vs. 66 MBq ^177^Lu |  | 1 | 0.882 | 0.142 |  |
| 1.0 MBq ^212^Pb vs. 22 MBq ^177^Lu |  | 1 | 0.005 | 0.002 |  |
| 1.0 MBq ^212^Pb vs. 43 MBq ^177^Lu |  | 1 | 1 | 0.057 |  |
| 1.0 MBq ^212^Pb vs. 66 MBq ^177^Lu |  | 1 | 1 | 0.464 |  |
| 0.24 MBq ^212^Pb vs. 0.5 MBq ^212^Pb |  | 1 | 1 | 1 | *  0.099 |
| 0.24 MBq ^212^Pb vs. 1.0 MBq ^212^Pb |  | 1 | 1 | 1 |  |
| 0.5 MBq ^212^Pb vs. 1.0 MBq ^212^Pb |  | 1 | 1 | 1 |  |
| 22 MBq ^177^Lu vs. 43 MBq ^177^Lu |  | 1 | 0.122 | 1 |  |
| 22 MBq ^177^Lu vs. 66 MBq ^177^Lu |  | 1 | 0.207 | 0.926 |  |
| 43 MBq ^177^Lu vs. 66 MBq ^177^Lu |  | 1 | 1 | 1 |  |

* The differences in the median values among the treatment groups are not great enough to exclude the possibility that the difference is due to random sampling variability; there is not a statistically significant difference.

**
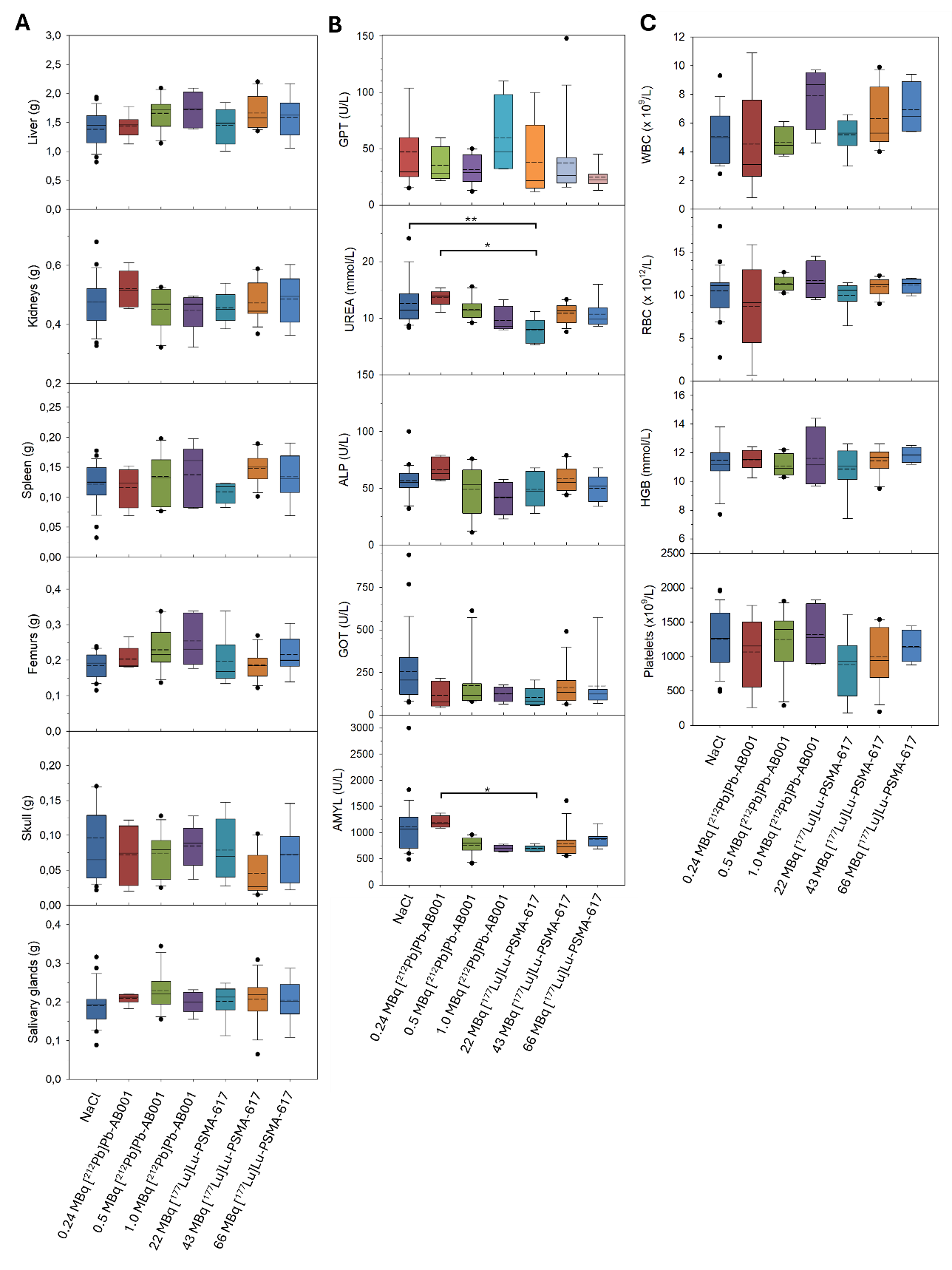
**

**Figure S7.** **Evaluation of physiological and biochemical parameters at the humane endpoint.** (A) Organ weights, (B) serum parameters and (C) haematological analysis sampled from mice at humane endpoint, treated with NaCl, 0.24 MBq [^212^Pb]Pb-AB001, 0.49 MBq [^212^Pb]Pb-AB001, 1.0 MBq [^212^Pb]Pb-AB001, 22 MBq [^177^Lu]Lu-PSMA-617, 43 MBq [^177^Lu]Lu-PSMA-617 or 66 MBq [^177^Lu]Lu-PSMA-617. The bottom of the boxes represents the 25th percentiles, solid lines represent the medians, dashed lines represent the mean, the top of the boxes represent the 75th percentiles and whiskers represent the 5th and 95th percentiles. One Way ANOVA was performed to compare statistical differences between groups for each measured parameter (*p = 0.01, **p = 0.002).

**Supplementary Discussion**

**Table S4. Therapeutic effect of various PSMA-targeting radioligands in metastatic prostate cancer models.** N/A, not available; IC, intracardiac; IV, intravenous; pci; post cell inoculation; TI, therapeutic index (median survival of treatment group divided by the median survival of the control group), p-value towards control.

| **Radioligand** | **Mouse strain** | **Cell line (injection route)** | **Injected activity  (time of treatment)** | **TI (P-value)** | **Reference** |
| --- | --- | --- | --- | --- | --- |
| [^211^At]At-6 | NOG | PC3-ML-Luc-PSMA (IV) | 0.11, 0.19, or 0.37 MBq (1 day pci) | 1.2, 1.8, 1.9 (p<0.01) | Kiess et al., 2016 [1] |
| [^211^At]At-3-Lu | NSG | PC3-ML-Luc-PSMA (IV) | 1.5 or 3.7 MBq  (1 week pci) | 1.2 (p = 0.03), 1.2 p = 0.03) | Mease et al., 2022 [2] |
| [^212^Pb]Pb-L2 | NSG | PC3-ML-Luc-PSMA (IV) | 3.7 MBq (1 day pci) | 1.3 (p < 0.01) | Banerjee et al., 2020 [3] |
| [^212^Pb]Pb-AB001 | Athymic nude | PC-3 PIP-luc (IC) | 0.24, 0.5, 1.0 MBq  (1 week pci) | 1.7, 1.7, 1.9 (p < 0.003) | Current study |
| [^177^Lu]Lu-PSMA-617 | Athymic nude | PC-3 PIP-luc (IC) | 22, 43, 66 MBq  (1 week pci) | 0.8, 1, 1.0  (p > 0.1) | Current study |
|  | NCG | C4-2 (IC) | 30 MBq (3 days pci) | 1.3 (N/A) | Lückerath et al., 2020 [4] |
|  | NSG | PC3-ML-Luc-PSMA (IV) | 37 MBq (1 day pci) | 1.0 | Banerjee et al., 2020 [3] |
|  | NSG | C4-2 (I.C.) | 35 MBq  (3 or 5 weeks pci) | 1.1 (p = 0.3), 1.3 (p < 0.01) | Meyer et al., 2023 [5] |
| [^177^Lu]Lu-L1 | NSG | PC3-ML-Luc-PSMA (IV) | 37 MBq (1 day pci) | 1.0 | Banerjee et al., 2021 [6] |
| [^225^Ac]Ac-L1 | NSG | PC3-ML-Luc-PSMA (IV) | 37, 74, 93, 4 × 9.3, 6 × 9.3, 8 × 9.3 kBq  (1 day pci) | 1.2 (p < 0.01), 1.7 (p < 0.01), 1.2 (N/A), 1.2 (N/A), 1.6  (p < 0.02), <1 | Banerjee et al., 2021 [6] |
| [^225^Ac]Ac-PSMA-617 | NSG | C4-2 (IC) | 40 kBq  (1 or 3 weeks pci) | 3.9 (p < 0.01), 1.9 (p < 0.01) | Stuparu et al., 2020 [7] |
|  | NSG | C4-2 (IC) | 40 kBq  (3 or 5 weeks pci) | 1.8 (p < 0.01), 1.8 (p < 0.01) | Meyer et al., 2023 [5] |
| [^177^Lu]Lu-PSMA-617 and [^225^Ac]Ac-PSMA-617 (tandem) | NSG | C4-2 (IC) | 17 MBq Lu + 20 kBq Ac  (3 or 5 weeks pci) | 1.7 (p < 0.01), 1.7 (p < 0.01) | Meyer et al., 2023 [5] |

**Supplementary References**

1. Kiess AP, Minn I, Vaidyanathan G, Hobbs RF, Josefsson A, Shen C, et al. (2S)-2-(3-(1-Carboxy-5-(4-211At-Astatobenzamido)Pentyl)Ureido)-Pentanedioic Acid for PSMA-Targeted α-Particle Radiopharmaceutical Therapy. J Nucl Med. 2016;57:1569-75. doi:10.2967/jnumed.116.174300.

2. Mease RC, Kang CM, Kumar V, Banerjee SR, Minn I, Brummet M, et al. An Improved (211)At-Labeled Agent for PSMA-Targeted α-Therapy. J Nucl Med. 2022;63:259-67. doi:10.2967/jnumed.121.262098.

3. Banerjee SR, Minn I, Kumar V, Josefsson A, Lisok A, Brummet M, et al. Preclinical Evaluation of (203/212)Pb-Labeled Low-Molecular-Weight Compounds for Targeted Radiopharmaceutical Therapy of Prostate Cancer. J Nucl Med. 2020;61:80-8. doi:10.2967/jnumed.119.229393.

4. Lückerath K, Bailis J, Current K, Salvati M, Radu C, Czernin J. 717 AMG 160, a prostate-specific membrane antigen (PSMA)-targeted BiTE® immuno-oncology therapy, is active in models of advanced prostate cancer that are resistant to radioligand therapy. Journal for ImmunoTherapy of Cancer. 2020;8:A429-A30. doi:10.1136/jitc-2020-SITC2020.0717.

5. Meyer C, Stuparu A, Lueckerath K, Calais J, Czernin J, Slavik R, Dahlbom M. Tandem Isotope Therapy with (225)Ac- and (177)Lu-PSMA-617 in a Murine Model of Prostate Cancer. J Nucl Med. 2023;64:1772-8. doi:10.2967/jnumed.123.265433.

6. Banerjee SR, Lisok A, Minn I, Josefsson A, Kumar V, Brummet M, et al. Preclinical Evaluation of (213)Bi- and (225)Ac-Labeled Low-Molecular-Weight Compounds for Radiopharmaceutical Therapy of Prostate Cancer. J Nucl Med. 2021;62:980-8. doi:10.2967/jnumed.120.256388.

7. Stuparu AD, Meyer CAL, Evans-Axelsson SL, Lückerath K, Wei LH, Kim W, et al. Targeted alpha therapy in a systemic mouse model of prostate cancer - a feasibility study. Theranostics. 2020;10:2612-20. doi:10.7150/thno.42228.
